# Achieving Decentralized, Electrified, and Decarbonized Ammonia Production

**DOI:** 10.1021/acs.est.3c10751

**Published:** 2024-04-11

**Authors:** Carlos A. Fernández, Oliver Chapman, Marilyn A. Brown, Christian E. Alvarez-Pugliese, Marta C. Hatzell

**Affiliations:** † George W. Woodruff School of Mechanical Engineering, Georgia Institute of Technology, Atlanta, Georgia 30318, United States; ‡ School of Public Policy, Georgia Institute of Technology, Atlanta, Georgia 30332, United States; § Department of Chemical Engineering, Texas Tech University, Lubbock, Texas 79409, United States; ∥ School of Chemical and Biomolecular Engineering, Georgia Institute of Technology, Atlanta, Georgia 30318, United States

**Keywords:** electrified chemical manufacturing, ammonia production, variable renewable electricity, decarbonization, geospatial optimization, water stress mitigation, decentralized supply chain, techno-economic modeling

## Abstract

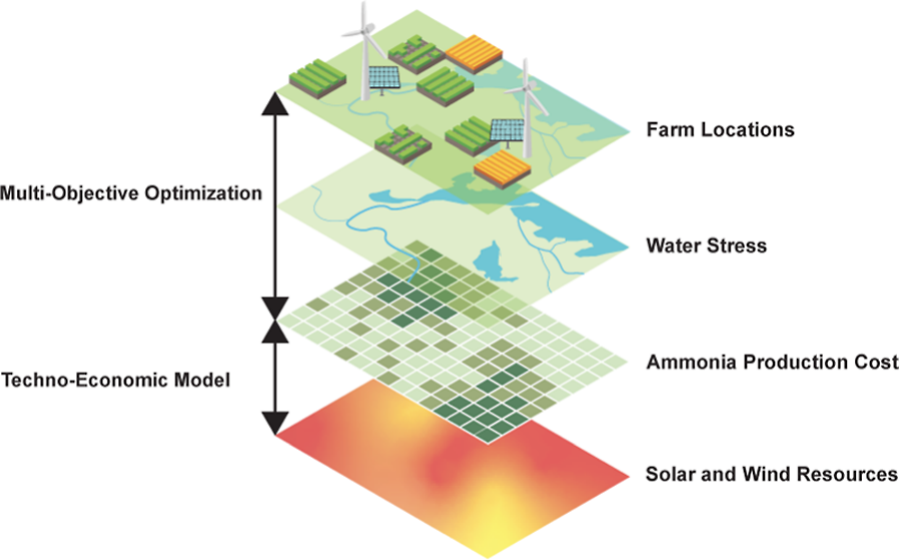

The rapid reduction in the cost of renewable energy has motivated the transition from carbon-intensive chemical manufacturing to renewable, electrified, and decarbonized technologies. Although electrified chemical manufacturing technologies differ greatly, the feasibility of each electrified approach is largely related to the energy efficiency and capital cost of the system. Here, we examine the feasibility of ammonia production systems driven by wind and photovoltaic energy. We identify the optimal regions where wind and photovoltaic electricity production may be able to meet the local demand for ammonia-based fertilizers and set technology targets for electrified ammonia production. To compete with the methane-fed Haber–Bosch process, electrified ammonia production must reach energy efficiencies of above 20% for high natural gas prices and 70% for low natural gas prices. To account for growing concerns regarding access to water, geospatial optimization considers water stress caused by new ammonia facilities, and recommendations ensure that the identified regions do not experience an increase in water stress. Reducing water stress by 99% increases costs by only 1.4%. Furthermore, a movement toward a more decentralized ammonia supply chain driven by wind and photovoltaic electricity can reduce the transportation distance for ammonia by up to 76% while increasing production costs by 18%.

## Abstract 2

Global fertilizer imbalances stem from the reliance on fossil fuel-driven ammonia production. This study proposes decentralized and sustainable ammonia production using wind and photovoltaic energy, aiming for an equitable and decentralized supply chain.

## Introduction

The ammonia produced by the Haber–Bosch process is essential for global agriculture as ammonia-based fertilizers supply more than half of the nitrogen demand required for plant growth.^1^ However, approximately 175 million tons of ammonia is produced yearly in fewer than 100 locations around the world.^2−6^ This results in transportation distances of thousands of kilometers in some instances. A consequence of centralized fertilizer manufacturing is that transportation costs can contribute more than 30% of the total fertilizer cost in certain regions.^3,4,7−9^ The cost of ammonia-based urea can range from as low as 300 USD/t in the United States to as high as 960 USD/t in Mozambique.^10^ This cost divergence often contributes to the underuse of synthetic ammonia-based fertilizers in many regions with growing populations, raising concerns for equitable development.^11^

Haber–Bosch facilities are also highly dependent on fossil fuels and produce 1.2% of global anthropogenic CO_2_ emissions and consume 2% of global energy.^5,6,12−14^ Reliance on fossil fuels contributes to unstable ammonia prices. Between 2020 and 2023, the average global cost of ammonia fluctuated between 400 USD/t_NH_3__ and 1600 USD/t_NH_3__, largely due to the volatility of natural gas prices in Europe and restrictions placed on Russian oil and natural gas.^15^ All of these challenges with modern ammonia manufacturing, inequity, lack of access, price volatility, and carbon emissions have motivated interest in exploring decarbonized and decentralized approaches for fertilizer production.^16−23^

Here, we perform a techno-economic analysis that takes into consideration geospatial data sets to assess the feasibility of ammonia production systems driven by wind and photovoltaic electricity. We compare the cost of electrified Haber–Bosch facilities to those of more emerging electrochemical technologies. When determining the spatial distribution of costs, we also take into consideration key technical, economic, and environmental conditions that can impact wind and photovoltaic electricity-driven ammonia production technologies. Then, we set energy efficiency and capital cost targets to meet the ammonia market prices. Finally, we optimize the ammonia production infrastructure driven by wind and photovoltaic electricity to minimize the impact that changes in transportation costs have on the cost of ammonia while also ensuring that ammonia production does not contribute to regional water stress.

The contribution of our study over existing research centers around the integration of a comprehensive techno-economic model with a distribution optimization model. By considering the spatial availability of resources such as land, water, and renewable energy sources like solar and wind, we offer an approach that addresses the intricate interplay between environmental constraints and sustainability research; building infrastructure that does not compete with land used for urban or industrial centers, land that is protected by national parks, or land that is situated in remote or inaccessible terrain. Additionally, building ammonia infrastructure that mitigates water stress ensures sustainable chemical manufacturing and the resiliency of the surrounding water systems. Our framework not only enhances the understanding of the economic viability of renewable electricity-driven ammonia production processes but also lays the groundwork for informed decision-making in transitioning toward sustainable practices. Moreover, incorporating distribution optimization models allows for the examination of the effect of decentralization on sustainable chemical manufacturing infrastructure.

## Methods

### General Methodological Framework

We use Aspen Plus to model the system’s mass and energy balances and to appropriately size each component. We developed a techno-economic model in Python for each system to estimate CapEx, OpEx, and ammonia production costs. These models take into consideration geospatial resource availability (solar, wind, water, and land) to calculate the geospatial distribution of ammonia production costs. Finally, we pair the techno-economic model with a distribution optimization algorithm to optimize the locations of ammonia production facilities for different technology and economic scenarios to reduce cost and water stress and improve resiliency.

### Process Description

Given the diversity and varying readiness levels of these electrified methods, we devised two models (Figure 2a,b). The first model describes the geospatial distribution of ammonia production costs of an electrified Haber–Bosch process consisting of a pressure swing adsorption air separation unit for nitrogen production, a water electrolyzer for hydrogen production, and a Haber–Bosch loop for ammonia production and purification (Figure 2a). This model draws upon the advanced readiness levels of each individual technology, incorporating descriptive ASPEN Plus models for each subsystem, component sizing, and capital cost (CapEx) calculations. Furthermore, a second model describes the geospatial distribution of ammonia production costs for a ‘Black Box’ electrochemical ammonia production system (Figure 2b). This ‘Black Box’ model consists of a pressure swing adsorption air separation unit for nitrogen production and a technology-agnostic model for ammonia production. This ‘Black Box’ model is based on a general model informed by projected capital cost and energy efficiency values. As such, this model can be applied to a wide variety of technologies that are currently under development.

### Technology Scenarios

An analysis of 12 technology development scenarios evaluates the economic feasibility of wind and photovoltaic electricity-driven ammonia production. The technology targets set here are for an electrified Haber–Bosch process and an electrochemical ‘Black Box’ process. The electrified Haber–Bosch process model examines three scenarios with varying water electrolyzer, wind, and solar installed capital costs. In contrast, the ‘Black Box’ model examines technologies with varying energy efficiencies, electrolyzer installed capital costs, PV installed capital costs, and wind installed capital costs. Note that the capital cost scenarios are based on the projected cost for electrolysis, photovoltaics, and wind technologies in 2050. The capital cost and energy efficiency scenarios are outlined in Table 1. There are alternative frugal approaches that may result in significantly lower capital costs; however, these are not considered due to the early stages of development.^4^

**Table 1:** Technology Scenarios

capital cost scenarios (USD/kW)			
parameter	low	medium	high
electrolyzer CapEx	200	550	900
PV CapEx	460	767	1322
wind CapEx	676	1127	1411

energy efficiency scenarios (%)			
parameter	low	medium	high
energy efficiency	20	40	60

### General Techno-Economic Model

The ammonia production cost, or levelized cost of ammonia, is a function of the discounted sum of the yearly costs over the discounted sum of the yearly ammonia produced across the lifetime of the project. The ammonia production cost (levelized cost of ammonia) can be calculated using eq 1.
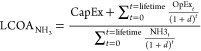
1where CapEx value is the initial capital investment, OpEx value is the yearly operation costs, *d* value is the discount rate, *t* value is the year, and NH_3_*t*__ value is the yearly ammonia production.

### General Distribution Optimization Model

To calculate the optimal distribution network, we used an exhaustive search algorithm that aims to minimize a score function (eq 2). This means that for every farm, we surveyed every possible ammonia production location and selected the one that resulted in the lowest score.
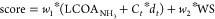
2where LCOA_NH_3__ is the ammonia production cost (i.e., levelized cost of ammonia) at the production location, *C*_*t*_ is the ammonia transportation cost in USD/ton_NH_3__-km, *d*_*t*_ is the transportation distance between the production facility and the farms, WS is the water stress at the production location, *w*_1_ is the weight placed on the ammonia cost, and *w*_2_ is the weight placed on the water stress. These weights signify the relative importance placed on cost and water. For example, a scenario that prioritizes cost has weights *w*_1_ equal to one and *w*_2_ equal to zero. This means that all of the importance is placed on minimizing the cost. On the other hand, a scenario that prioritizes water has weights *w*_1_ = 0.01 and *w*_2_ = 0.99. This means that the optimization score is composed of 1% by the ammonia cost and 99% by the water stress. The exhaustive optimization algorithm minimizes the score in eq 2 for every possible farm to find the optimal production location for all farms. The transportation cost (*C*_*t*_) is assumed to be 0.016 USD/ton_NH_3__-km for transportation by ship, 0.04 USD/ton_NH_3__-km for transportation by pipeline, and 0.09 USD/ton_NH_3__-km for transportation by truck.^24^ For the baseline scenario, we used a transportation cost of 0.09 USD/ton_NH_3__-km. Finally, the distribution distance (*d*_*t*_) between the production facilities and the farms is calculated using the haversine formula (eq 3).
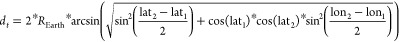
3where *R*_Earth_ is the radius of the earth in kilometers (*R*_Earth_ = 6373 km), lat_1_ and lon_1_ are the coordinates of the prospective production location, and lat_2_ and lon_2_ are the coordinates of the farm. A complete explanation of the method can be found in the Supporting Information.

## Results and Discussion

### Projections of the Ammonia Production Cost for the Methane-Fed Haber–Bosch Process

**Figure 1:**
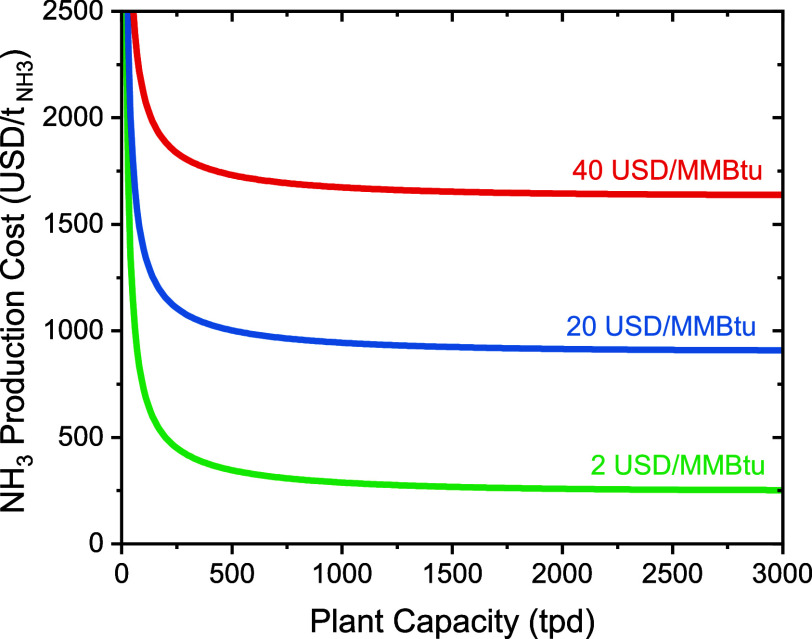
Methane-fed Haber–Bosch process production cost as a function of plant capacity.

The current methane-fed Haber–Bosch process produces ammonia in a centralized manner. Haber–Bosch facilities are currently built in locations that have access to natural gas and are close to chemical industrial centers. The cost of ammonia production today is heavily influenced by the scale of production and the price of natural gas (Figure 1). Smaller production scales result in higher production costs due to limited economies of scale, equipment costs, and labor costs that do not decrease proportionately with scale. Ideally, Haber–Bosch facilities operate on production scales in the range of thousands of metric tons per day, enabling them to achieve production costs as low as 250  when natural gas prices are low (∼2 USD/MMBtu). However, as production scales decrease (∼50 tpd), the production cost can increase by more than five times to 1300 . Moreover, even at large production scales, the production cost of ammonia is highly sensitive to natural gas prices. In early 2022, natural gas prices went from around 2 to over 40 USD/MMBtu. Even at large production scales (∼2500 tpd), the ammonia production cost increases from 250  to 1600  as the natural gas price increases from 2 USD/MMBtu to 40 USD/MMBtu, respectively.

Given the centralized nature of the Haber–Bosch process, there are fewer than a hundred production facilities worldwide. These facilities are predominantly located in high-income countries that have access to inexpensive natural gas and advanced chemical infrastructure. The average distance between the Haber–Bosch facilities and farms is around 1200 km. However, it is important to note that despite the presence of nearby Haber–Bosch facilities, certain regions in the world still struggle to meet their regional demand for ammonia. This can be attributed to various factors, such as limited infrastructure, inadequate access to resources, or economic constraints. In such cases, the proximity of centralized production facilities may not be sufficient to address the specific regional needs. Thus, there is an increasing interest in exploring alternative production and distribution models that incorporate decentralization, renewable energy sources, and regional production centers that can effectively cater to the demands of these underserved regions. Such approaches aim to address the challenges associated with cost fluctuations and create a more sustainable and resilient ammonia supply chain.

### Projections of the Cost of Wind and Photovoltaic Electricity-Driven Ammonia Production

**Figure 2:**
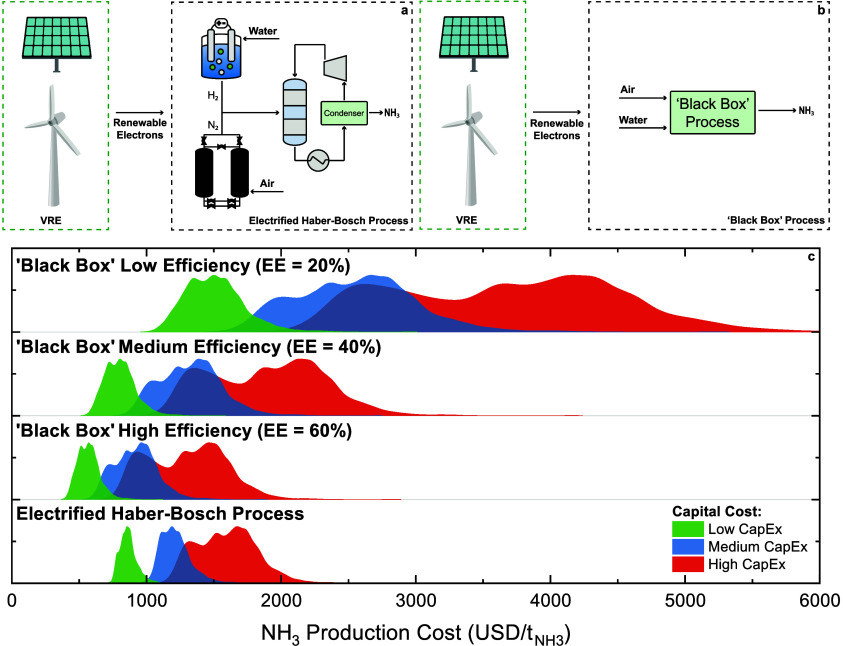
System diagram for an electrified Haber–Bosch process powered by wind and photovoltaic electricity (a), system diagram for an electrochemical ‘Black Box’ process powered by wind and photovoltaic electricity (b), and geospatial distribution of wind and photovoltaic electricity-driven ammonia production costs (c). The *y* axis in (c) corresponds to the density of the cost data being depicted in the *x* axis.

Over the past decade, rapid advances to decarbonize ammonia production have focused on replacing steam methane reforming with water electrolysis prior to the Haber–Bosch process (Figure 2a) and on developing electrochemical pathways for ammonia synthesis (Figure 2b).^12,13,18,25^ These electrochemical pathways include the direct electrochemical nitrogen reduction reaction, lithium-mediated approaches, and plasma-assisted approaches.^26−30^ The ammonia production cost distribution provides valuable insights into the economic viability of each development scenario (Figure 2c). When the interaction between energy efficiency and capital costs is considered, the analysis offers critical information for decision-making in the development of technologies for ammonia production.

The electrified Haber–Bosch process is used as a decarbonized baseline to the electrochemical ‘Black Box’ process due to its advanced readiness level. In the electrified Haber–Bosch process, the average geographic cost of ammonia production varies widely, from 870  in the low-capital cost scenario to 1600  in the high-capital cost scenario (Figure 2c). Interestingly, the lower end of this range is nearly half of the highest market prices observed in recent years (∼1600  — July 2022), yet nearly two times higher than the lowest market prices observed in recent years (∼400  — September 2020). Therefore, sustained high natural gas prices are necessary for the electrified Haber–Bosch process to achieve price parity with the methane-fed Haber–Bosch process. Natural gas prices must remain above 18 USD/MMBtu for the low-cost scenario to be feasible and above 39 USD/MMBtu for the high-cost scenario to be feasible.

Pivoting to electrochemical ‘Black Box’ systems, which allows us to study the behavior of technologies that are in earlier stages of development delineated by their respective energy efficiencies—low-efficiency (EE = 20%),^31−37^ medium-efficiency (EE = 40%),^38−41^ and high-efficiency (EE = 60%). For low-efficiency systems, the average geographic cost of ammonia production varied from 1500  in the low-capital cost scenario to 3700  in the high-capital cost scenario (Figure 2c). The lower end of this range is comparable to the highest market prices observed in recent years and is nearly four times higher than the lowest market prices observed in recent years. Subsequently, for medium-efficiency systems, the average geographic cost of ammonia production varied from 800  in the low-capital cost scenario to 1900  in the high-capital cost scenario. The lower end of this range is half of the highest market prices observed in recent years and is nearly two times higher than the lowest market prices observed in recent years. Finally, for high-efficiency systems, the average geographic cost of ammonia production varied from 570  in the low-capital cost scenario to 1300  in the high-capital cost scenario. The lower end of this range is a third of the highest market prices observed in recent years and comparable to the lowest market prices observed in recent years.

The natural gas prices required for ‘Black Box’ systems to reach price parity with the methane-fed Haber–Bosch process vary greatly depending on the energy efficiency of the system. For low-efficiency systems, natural gas prices must remain above 36 USD/MMBtu for the low-cost scenario to be feasible and above 90 USD/MMBtu for the high-cost scenario to be feasible. Additionally, for medium-efficiency systems, natural gas prices must remain above 17 USD/MMBtu for the low-cost scenario to be feasible and above 47 USD/MMBtu for the high-cost scenario to be feasible. Finally, for high-efficiency systems, natural gas prices must remain above 10 USD/MMBtu for the low-cost scenario to be feasible and above 30 USD/MMBtu for the high-cost scenario to be feasible. Our results indicate that low-efficiency ‘Black Box’ systems are only feasible if natural gas prices return to an all-time high, medium-efficiency ‘Black Box’ systems are a viable competitor to the Haber–Bosch only if natural gas prices remain volatile, and high-efficiency ‘Black Box’ systems could compete with the Haber–Bosch process even if natural gas prices decrease from current levels.

Our analysis suggests a clear correlation between the viability of ammonia production technologies, both the electrified Haber–Bosch and the electrochemical “Black Box” systems, and parameters such as energy efficiency, capital costs, and natural gas prices. The electrified Haber–Bosch process emerges as a decarbonized alternative that can reach cost competitiveness under high natural gas price conditions. Conversely, electrochemical ‘Black Box’ technologies, with their higher uncertainty, reveal that high-efficiency systems can compete with traditional methods even under lower natural gas prices, whereas low- and medium-efficiency systems are only viable with sustained high natural gas prices. These results highlight the need for enhancing the energy efficiency and reducing capital costs to improve the economic viability and environmental benefits of ammonia production technologies. Furthermore, these results emphasize the significance of the natural gas market conditions in determining the success of these decarbonized approaches to ammonia production. The fluctuating prices of natural gas play a fundamental role in the competitiveness of these technologies, underlining the interconnectedness of energy markets with the adoption and scalability of sustainable solutions for ammonia manufacturing.

### Optimizing Production and Distribution Networks for Wind and Photovoltaic Electricity-Driven Ammonia Production

**Figure 3:**
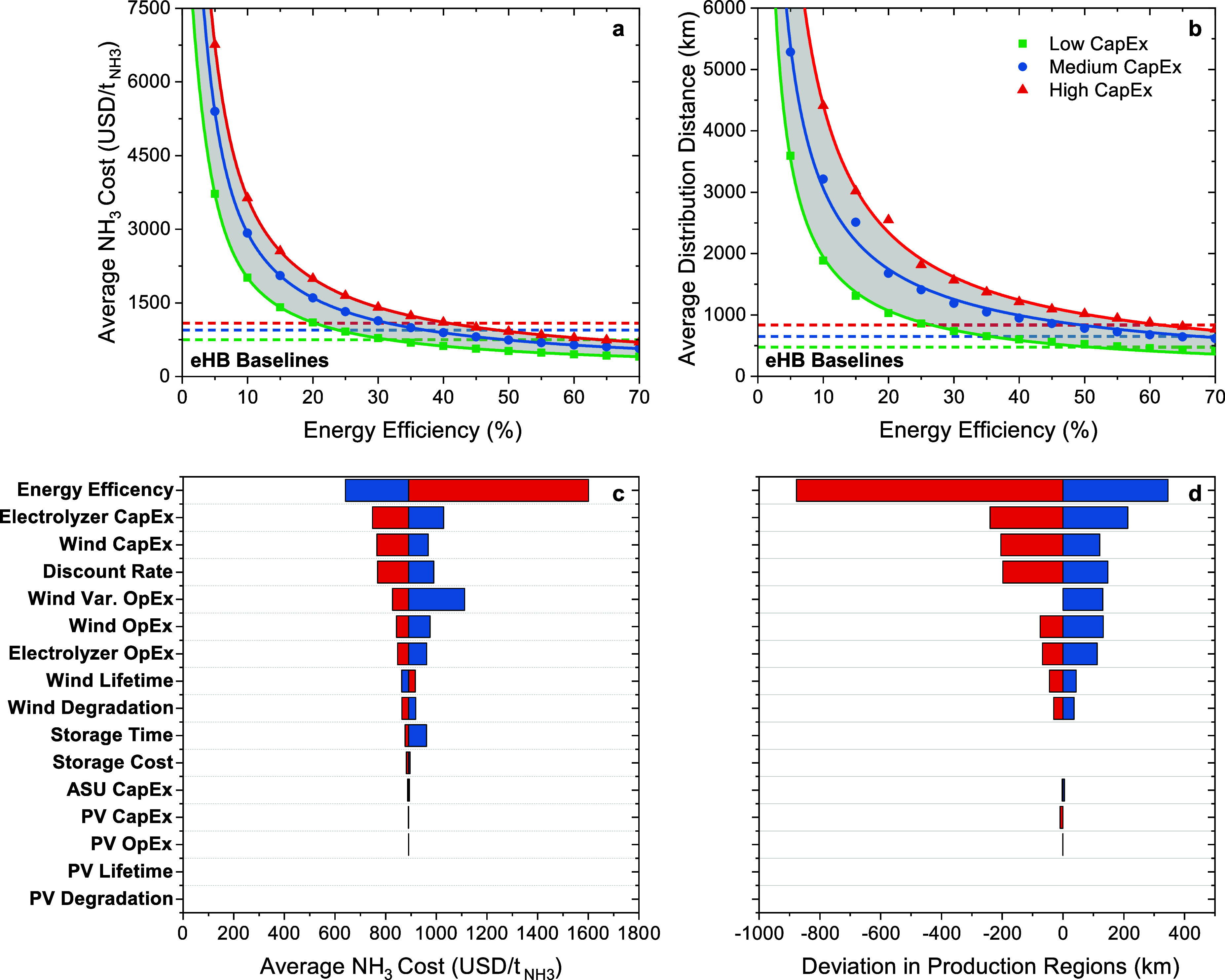
Average ammonia cost (production + distribution) for an optimized production and distribution network (a) and average distribution distance for an optimized production and distribution network (b). The horizontal dashed lines represent the electrified Haber–Bosch baseline for each of the capital cost scenarios. The production and distribution networks were optimized assuming an ammonia transportation cost of 0.09 -km and the capital cost scenarios are outlined in Table 1. Sensitivity analysis for the average ammonia production cost for an optimized production and distribution network (c) and optimal production region location for an optimized production and distribution network (d). Relevant parameters for the sensitivity analysis are shown in Table S3.

Expanding the previous analysis to optimize the production and distribution networks highlights the potential for implementing each technology scenario. For the electrified Haber–Bosch process, our model suggests an optimal setup of 78 regional production locations worldwide under the high-cost scenario and 144 regional production locations worldwide under the low-cost scenario. Here, the average production cost within an optimized network ranges from 707  in the low-cost scenario to 1015  in the high-cost scenario (Figure 3c). Similarly, transportation costs vary between 43  in the low-cost scenario and 75  in the high-cost scenario (Figure 3a), with average transportation distances spanning from 480 to 840 km, respectively (Figure 3b).

These findings indicate that an electrified Haber–Bosch process, powered by wind and photovoltaic energy, could offer a viable alternative to the traditional methane-fed Haber–Bosch process if the costs of wind turbines, photovoltaic cells, and electrolyzers decrease in the following decades. Additionally, our results highlight the logistical advantages of the regional distribution seen in our models in reducing distribution costs and improving the regional availability of ammonia. Several analyses concluded that electrifying the Haber–Bosch process could reduce carbon emissions from 1.7 / to 0.5 /.^13^ As such, the shift toward electrification not only promises a reduction in carbon emissions but also challenges the conventional highly centralized production model by introducing the possibility of a more regional production network. However, these large-scale facilities are still unable to produce ammonia in a highly distributed manner at different production scales.^2,32,42,43^ This is an important distinction between the more conventional Haber–Bosch process-based approaches (Figure 2a) and the electrochemical ‘Black Box’ approaches examined next (Figure 2b).

For low-efficiency ‘Black Box’ systems, our model suggests an optimal setup of 124 regional production locations worldwide under the low-cost scenario and 32 regional production locations worldwide under the high-cost scenario. In this case, the average production cost for an optimized production and distribution network is between 1000  in the low-cost scenario and 1800  in the high-cost scenario (Figure 3a). Similarly, transportation costs vary between 90  in the low-cost scenario and 230  in the high-cost scenario (Figure 3a), with average transportation distances spanning from 1000 to 2500 km, respectively (Figure 3b). A technology that may operate at these energy efficiencies is a nitrogen electrolysis cell.^44^ Considering the low performance and nonideal centralized network, there are few gains from such a system.

For medium-efficiency ‘Black Box’ systems, our model suggests an optimal setup of 329 regional production locations worldwide under the low-cost scenario and 92 regional production locations worldwide under the high-cost scenario. This network size mirrors that of the Haber–Bosch process in terms of the degree of centralization. Here, the average production cost for an optimized production and distribution network is between 560  in the low-cost scenario and 1000  in the high-cost scenario (Figure 3a). Similarly, transportation costs vary between 55  in the low-cost scenario and 110  in the high-cost scenario (Figure 3a), with average transportation distances spanning from 600 to 1200 km, respectively (Figure 3b). While 92–329 facilities are still centralized, the ability to distribute these facilities across the globe, rather than clustering the facilities, reduces the distance between manufacturing locations and farms by nearly two times when compared to current Haber–Bosch facilities, which have an average distance between Haber–Bosch locations and farms of 1200 km. This would aid in increasing access and would potentially reduce safety issues. An emerging technology that may be able to operate at these energy efficiencies is lithium-mediated electrochemical nitrogen reduction.^45,46^

For high-efficiency ‘Black Box’ systems, our model suggests an optimal setup of 795 regional production locations worldwide under the low-cost scenario and 164 regional production locations worldwide under the high-cost scenario. Here, the average production cost for an optimized production and distribution network is between 410  in the low-cost scenario and 710  in the high-cost scenario (Figure 3a). Similarly, transportation costs vary between 41  in the low-cost scenario and 80  in the high-cost scenario (Figure 3a), with average transportation distances spanning from 460 to 880 km, respectively (Figure 3b)—which is up to three times lower than the minimum distance between Haber–Bosch locations and farms. Thus, high-efficiency wind and photovoltaic electricity-driven ammonia production systems are an essential requirement for the decentralized chemical manufacturing of fertilizers.

To achieve an ammonia cost (production + distribution) under the highest market price in the last 5 years (∼1600 ), wind and photovoltaic electricity-driven ammonia production technologies must achieve energy efficiencies above 25% in the high-cost scenario, energy efficiencies above 20% in the medium-cost scenario, or energy efficiencies above 15% in the low-cost scenario (Figure 3a). In contrast, to achieve an ammonia cost (production + distribution) under the lowest market price in the last 5 years (∼400 ), wind and photovoltaic electricity-driven ammonia production technologies must achieve energy efficiencies above 70% and only the low-cost scenario is viable (Figure 3a). Note, however, that these prices do not take into consideration environmental externalities, geopolitics, or government-based subsidies.

Therefore, improving the energy efficiency of state-of-the-art wind and photovoltaic electricity-driven ammonia production systems over selectivity is the critical performance metric in order to achieve decentralized wind and photovoltaic electricity-driven ammonia. On that account, it might be prudent to focus policy and investments in research and development while the energy efficiencies remain low (EE < 40%) and then transition policy-guided investments toward strategies to minimize ammonia costs through incentives, taxes, efficiency standards, and the scale-up of renewable ammonia production technologies. Finally, our results highlight the importance of codevelopment and free-trade strategies within neighboring countries to promote affordable and equitable wind and photovoltaic electricity-driven ammonia. Due to the variability of local climates, optimal regions for wind and solar do not always overlap existing arable land. Countries with wind and solar resources beyond their own agricultural needs (e.g., Botswana, Chile, Australia) may lack the resources or desire to build the wind and photovoltaic electricity-driven ammonia installations needed to meet the global demand for ammonia. These potential exporting countries could benefit from codevelopment strategies by sharing the costs of developing wind and photovoltaic electricity-driven ammonia resources with neighboring countries. In return, countries that lack solar and wind resources will benefit from lower import tariffs and discounted fertilizer prices, which will give them access to affordable wind and photovoltaic electricity-driven ammonia.

### Sensitivity Analysis for Wind and Photovoltaic Electricity-Driven Ammonia Production

A sensitivity analysis of the average ammonia production cost (Figure 3c) and the optimal production region locations for an optimized production and distribution network for a “Black Box” ammonia production system (Figure 3d) show the influence each technical and economic parameter has on the ammonia production cost and the optimal locations of wind and photovoltaic electricity-driven ammonia production regions. The average ammonia production cost in an optimized production and distribution network is significantly impacted by various critical parameters. Notably, the system’s energy efficiency, electrolyzer CapEx and OpEx, wind CapEx and OpEx, and discount rate exhibit the highest level of sensitivity. Improving the system’s energy efficiency from 40 to 60% leads to a 28% reduction in the average ammonia costs, whereas a decrease in energy efficiency from 40 to 20% results in an 80% increase in average ammonia costs. Variations in the electrolyzer capital cost introduce a 16% deviation in the average ammonia costs from the reference scenario. Similarly, an increase in the wind capital cost corresponds to a 9% increase in ammonia costs, while a reduction in the wind capital cost yields a 14% decrease in ammonia costs. Furthermore, variations in the discount rate also play a significant role, with a high discount rate (10%) causing an 11% increase and a low discount rate (3%) resulting in a 14% decrease in the average ammonia cost compared to the reference scenario (7%).

Similarly, the optimal locations for production regions are significantly impacted by the system’s energy efficiency, electrolyzer CapEx and OpEx, wind CapEx and OpEx, and discount rate. Improving the system’s energy efficiency from 40 to 60% results in a 345 km discrepancy in the optimal location for production regions, whereas a decrease in energy efficiency from 40 to 20% results in an 880 km discrepancy in the optimal location for production regions. Similarly, a high electrolyzer capital cost results in a 215 km discrepancy in the optimal location for production regions, and a low electrolyzer capital cost results in a 240 km discrepancy in the optimal location for production regions. Similarly, an increase in the wind capital cost corresponds to a 120 km discrepancy in the optimal location for production regions, while a reduction in the wind capital cost results in a 205 km discrepancy in the optimal location for production regions. Furthermore, variations in the discount rate also play a significant role, with a high discount rate (10%) causing a 150 km discrepancy in the optimal location for production regions and a low discount rate (3%) resulting in a 200 km discrepancy in the optimal location for production regions compared to the reference scenario (7%).

Surprisingly, the photovoltaic system’s economic parameters (CapEx and OpEx) exert minimal influence on the average ammonia production cost in the optimized production and distribution network and on the optimal locations for production regions. This trend can be attributed to the preference for wind energy over photovoltaic energy to power electrified ammonia production technologies. For a fixed ammonia production rate, wind energy offers advantages such as lower electricity costs and electrolyzer capital costs due to its higher capacity factor when compared with photovoltaic energy. The choice between wind and photovoltaic energy for powering electrified ammonia production technologies is influenced by the local availability of these renewable resources, rather than a universal preference for one over the other. Wind energy is preferred in regions in which wind resources are abundant and near agricultural centers. This is largely due to wind’s higher capacity factor, which allows for more consistent and efficient ammonia production from intermittent energy sources. Our analysis reveals that a majority of the optimized facilities for electrified ammonia production predominantly utilize wind energy, reflecting its significant role in the sensitivity of the model. To a lesser extent, in areas with high solar irradiance, photovoltaic energy becomes more favorable, contributing to diversity in energy sources. This variation is highlighted by the model’s sensitivity analysis and the observable trend toward solar energy in more decentralized systems (Figure 8). Therefore, the preference between wind and photovoltaic energy for electrified ammonia production is a reflection of the optimal utilization of local renewable resources, ensuring both the economic viability and environmental sustainability of the production process.

The most important parameters that govern the production cost and optimal locations for a ‘Black Box’ ammonia production system are the system’s energy efficiency, discount rate, electrolyzer CapEx and OpEx, and wind CapEx and OpEx. As the energy efficiency of a technology increases, the capacity of the system to operate in a decentralized manner increases. These parameters alter the geographic distribution of the ammonia production costs. As the production costs change, the optimal solution, which considers both production and distribution costs, also varies. The most cost-effective locations for production facilities also change to minimize the total cost of ammonia at each location. For example, an increase in energy efficiency leads to lower ammonia production costs with a narrower cost distribution (Figure 2c)—implying that the difference in production costs between low-cost and high-cost regions is smaller. This results in distribution costs having a greater impact on total costs, which promotes a more distributed production network of facilities located closer to agricultural centers.

### Wind and Photovoltaic Electricity-Driven Ammonia and Economic Indicators

An essential economic indicator for evaluating the economic feasibility of wind and photovoltaic electricity-driven ammonia production is the discount rate. The discount rate represents the rate of return used to evaluate the present value and the cash flow of a project. The chosen discount rate depends on the inflation rate, risk, and funding source, with government funding having lower discount rates than private funding. The determination of an appropriate discount rate is complex, requiring a holistic approach that considers market distortions caused by subsidies, the technicalities of integrating renewables into existing systems, and the social implications of community involvement.^47−49^ Moreover, it necessitates a comprehensive analysis of various factors, including interest rates, expected returns, the time frame of the analysis, and risk premiums, while also adapting to extraordinary conditions such as pandemics, global conflicts, climate issues, and other unique challenges associated with renewable energy projects. A higher discount rate reduces the present value of future cash flows, leading to more centralized systems that have lower capital costs but higher operating costs. A lower discount rate has the opposite effect, increasing the present value of future cash flows and leading to more decentralized systems that have higher capital costs but lower operating costs.

**Figure 4:**
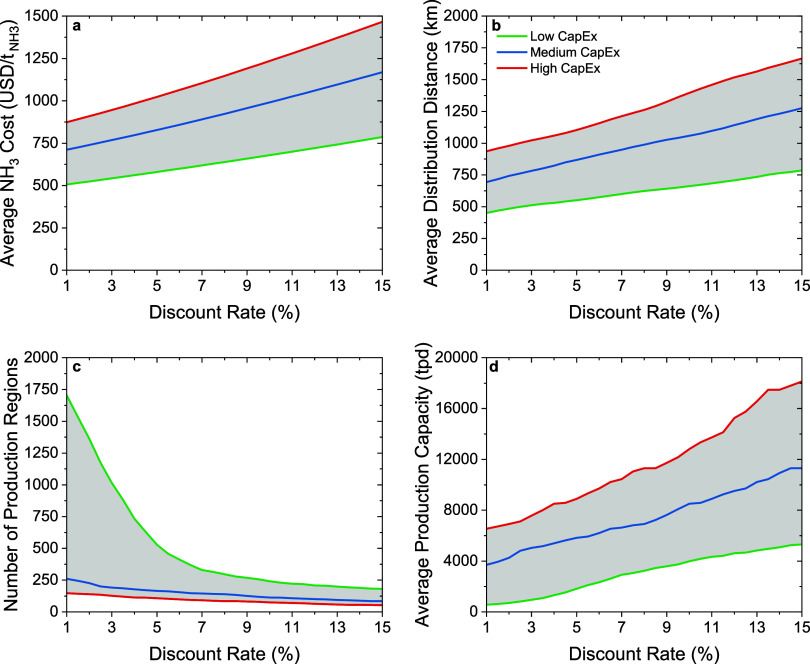
Effect of the discount rate on the average ammonia cost (a), average ammonia distribution distance (b), number of optimal production regions (c), and average regional production capacity (d). The production and distribution networks were optimized assuming an ammonia transportation cost of 0.09 -km for the medium-efficiency ‘Black Box’ system (EE = 40%).

An analysis of three discount rate scenarios evaluates the effect of the discount rate on the average ammonia cost, average distribution distance, optimal number of production regions, and average regional production capacity. A low discount rate, here considered to be 3%, results in an average ammonia cost of 540  in the low-cost scenario and 950  in the high-cost scenario (Figure 4a), with an average distribution distance of 510 and 1020 km, respectively (Figure 4b). Similarly, under a 3% discount rate, the optimal production and distribution network consists of 1015 production regions with an average capacity of 950 t of ammonia per day in the low-cost scenario and 127 production regions with an average capacity of 7600 t of ammonia per day in the high-cost scenario (Figure 4c,d).

A medium discount rate, here considered to be 7%, results in an average ammonia cost of 620  in the low-cost scenario and 1100  in the high-cost scenario (Figure 4a), with an average distribution distance of 600 and 1200 km, respectively (Figure 4b). Similarly, under a 7% discount rate, the optimal production and distribution network consists of 329 production regions with an average capacity of 2900 t of ammonia per day in the low-cost scenario and 92 production regions with an average capacity of 10,500 t of ammonia per day in the high-cost scenario (Figure 4c,d).

A high discount rate, here considered to be 10%, results in an average ammonia cost of 680  in the low-cost scenario and 1230  in the high-cost scenario (Figure 4a), with an average distribution distance of 660 and 1400 km, respectively (Figure 4b). Similarly, under a 10% discount rate, the optimal production and distribution network consists of 241 production regions with an average capacity of 4000 t of ammonia per day in the low-cost scenario and 75 production regions with an average capacity of 13,000 t of ammonia per day in the high-cost scenario (Figure 4c,d).

Our results suggest that higher discount rates lead to higher ammonia costs, higher distribution distances, higher average regional capacity, and a lower number of production regions. Conversely, lower discount rates result in lower ammonia costs, shorter distribution distances, smaller regional production capacities, and a higher number of production regions. Therefore, the selected discount rate is an important parameter in shaping strategies for decarbonizing and decentralizing ammonia production. On that regard, it is essential for governments and entities to provide funding programs with low discount rates for building renewable ammonia production infrastructure. These funding opportunities, having lower discount rates, would allow for lower ammonia costs and distribution distances and a more decentralized and resilient production and distribution network for wind and photovoltaic electricity-driven ammonia production systems. These conclusions serve as crucial insights for decision makers in the landscape of renewable-energy-driven ammonia production.

### Wind and Photovoltaic Electricity-Driven Ammonia and Water Uncertainty

A resource-related challenge to wind and photovoltaic electricity-driven ammonia production is the need for clean water as it requires a minimum of 1.6 t of water for each metric ton of ammonia produced.^17^ We must consider the spatial distribution of water stress to create an ammonia production infrastructure that is not affected by changes in seasonal water availability. To do this, we modified the optimization eq 2 by adding a weight (*w*_1_) to the ammonia production cost and a weight (*w*_2_) to the water stress at the possible production locations. By changing the values of *w*_1_ and *w*_2_, we can vary the relative importance placed on the ammonia cost and water stress.

**Figure 5:**
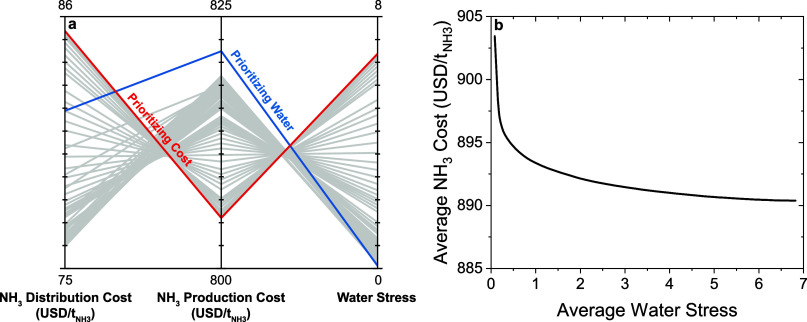
Effect of the importance placed on water stress on the average ammonia distribution cost, average ammonia production cost, and average water stress (a) and Pareto frontier representing the trade-off between ammonia cost and water stress (b). The production and distribution networks were optimized assuming an ammonia transportation cost of 0.09 -km for the medium cost scenario and a 40% energy efficiency. We study scenarios with varying values for *w*_1_ and *w*_2_ that are within these two scenarios (0.01 > *w*_1_ > 1, 0 > *w*_2_ > 0.99, and *w*_1_ + *w*_2_ = 1).

As the importance placed on water stress increases, the average water stress decreases dramatically (Figure 5a). The same increase in the importance placed on water stress leads to a marginal increase in ammonia production and distribution costs. For example, a scenario that prioritizes cost over water (Figure 5a—red line) results in an average ammonia production cost of 805 , a distribution cost of 85 , and a water stress of 6.8 (indicating that the water usage in the region is 6.8 times larger than the available water in the region). Therefore, placing no importance on water stress and prioritizing cost when building future wind and photovoltaic electricity-driven ammonia infrastructure could lead to further stress in regions where water scarcity is already an issue.

In contrast, a scenario that prioritizes water over cost (Figure 5a—blue line) results in an increase in the average ammonia production cost to 822 , a decrease in the distribution cost to 82 , and a decrease in the water stress to 0.08 (indicating that the water usage is 8% of the total available water). Therefore, placing more importance on water stress results in a 1.4% increase in the ammonia production and distribution cost and a 99% decrease in the average water stress. A Pareto frontier highlights the trade-off between cost and water stress in optimal solutions (Figure 5b). The marginal change in the average ammonia cost is inversely proportional to the average water stress. For instance, the initial 25% reduction in water stress (from 6.8 to 5.1) results in a 0.3  increase in the average ammonia cost. In contrast, the final 25% reduction in water stress (from 2 to 0.08) results in an 11  increase in the average ammonia cost. This represents a 40-time difference between the response of cost to changes in water stress in the final stages and the initial stages. In the highly competitive ammonia and fertilizer industries, even a small change in production costs, such as a 0.3  or 11  increase due to minimizing the regional water stress, can have substantial implications for revenue. Given the massive volumes of ammonia produced, even marginal cost changes are amplified across millions of tons, leading to a significant financial impact. Producers may not always have the flexibility to pass these cost increases onto customers due to competitive market pricing or fixed contractual agreements, which could force them to absorb these costs, directly cutting their revenue margins. These results highlight the trade-off in resource management and cost, emphasizing the importance of site selection to minimize water stress while keeping costs low.

**Figure 6:**
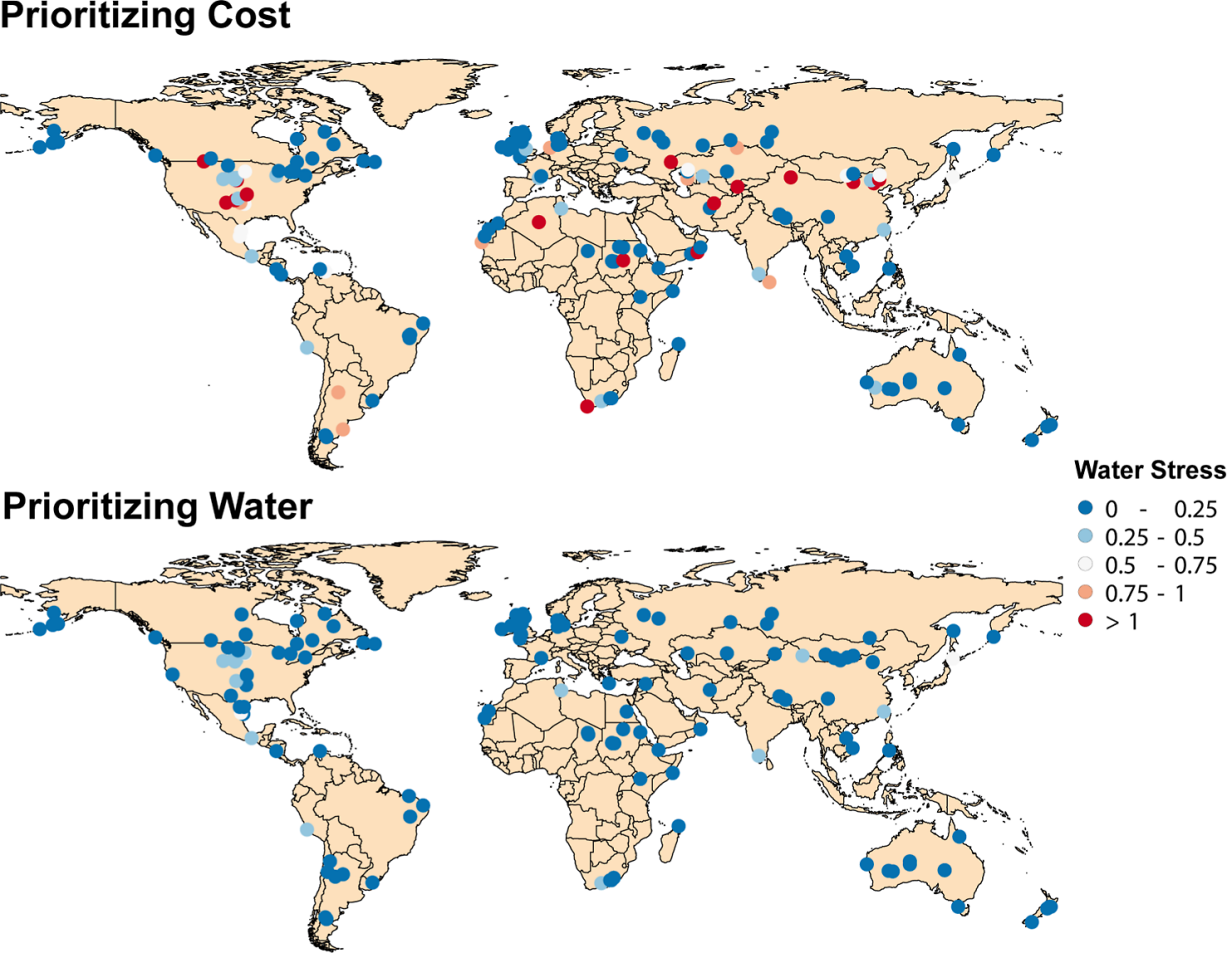
Optimal location for ammonia production facilities for a scenario prioritizing cost and a scenario prioritizing water. The production and distribution networks were optimized assuming an ammonia transportation cost of 0.09 -km for the medium-cost scenario and a 40% energy efficiency.

The drastic reduction in the average water stress without sacrificing cost is possible due to a slight rearrangement of the location of the ammonia production facilities (Figure 6). This analysis shows a pathway to building a wind and photovoltaic electricity-driven ammonia production infrastructure without exacerbating regional water stress. This is essential to prevent water consumption for ammonia production from competing with water consumption for public supply, irrigation, and power generation in already water-depleted regions. Our results can benefit regions with high levels of wind, solar irradiance, and water stress, such as the southwest of the United States, Sub-Saharan Africa, and regions of central and east Asia (Figure 6).

In the scenario prioritizing cost over water, the optimization model is insensitive to water stress and optimizes solely on the basis of cost. Therefore, optimal locations for production facilities (Figure 6) have an average water stress of 6.8, and several facilities are located in regions with water stress above one, meaning that these facilities are in regions that consume more water than what is available in the region. Thus, these facilities will not have access to a reliable supply of fresh water. In the scenario prioritizing water, all facilities have water stress under 0.5, with an average of 0.08 (Figure 6). The location of the facilities does not drastically change between scenarios because the facilities migrate from locations with the best wind and solar resources, but poor water availability, to adjacent regions that have excellent wind and solar resources, but not the best, and access to a reliable source of water. The water stress of adjacent locations could differ due to the local effects of population and industrial water usage or the proximity to bodies of water. This study focuses on geographical optimization to mitigate water stress in ammonia production without delving into specific process optimization for water use reduction. However, we acknowledge that policies promoting water-efficient technologies and technical measures, such as closed-loop systems, could further enhance water sustainability in these regions.

### Wind and Photovoltaic Electricity-Driven Ammonia and Distribution Uncertainty

With increasingly uncertain oil prices and global distribution systems, the ammonia production infrastructure must be insensitive to changes in ammonia transportation costs.^50^ The need for a more robust chemical supply chain has become evident with the increase in distribution costs and supply chain issues in the last 2 years. A decentralized production infrastructure results in shorter transportation distances and therefore lower distribution costs. Furthermore, decentralized production could lead to improved resiliency to the failure of production nodes.

**Figure 7:**
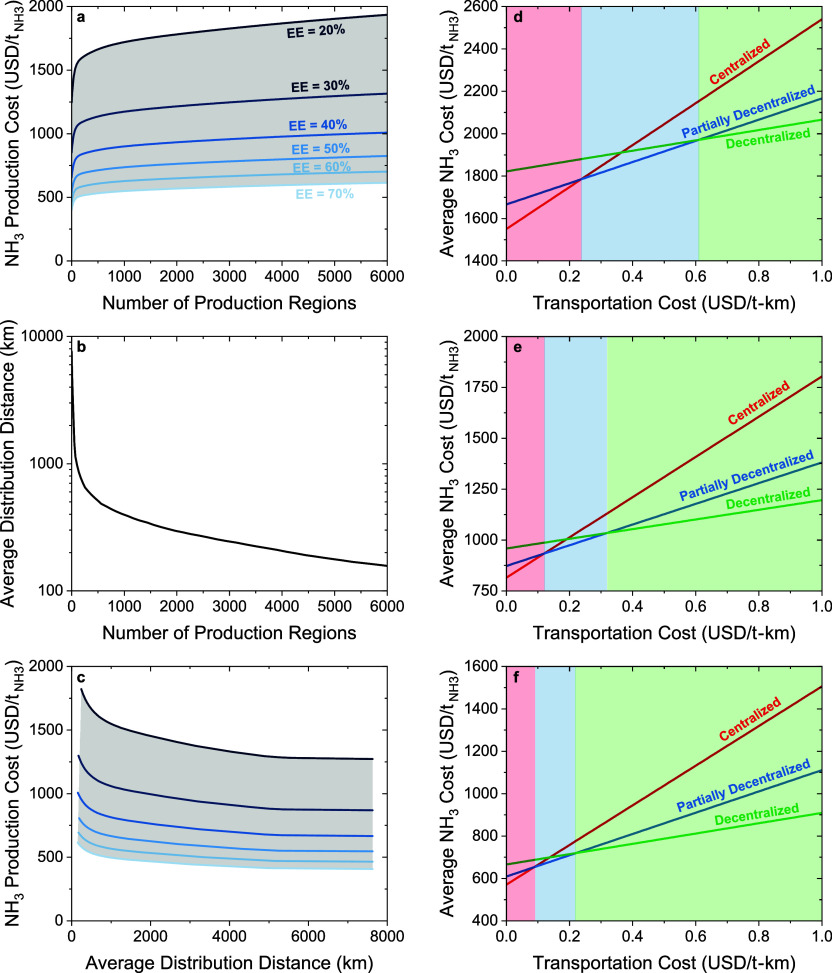
Effect on number of production regions on the ammonia production cost (a), distribution distance (b), and Pareto frontier representing the trade-off between ammonia production cost and distribution distance (c) for the medium capital cost scenario. Variation of the ammonia cost (production + distribution) with changes in transportation costs for centralized, partially decentralized, and fully decentralized scenarios for systems with 20% energy efficiency (d), 40% energy efficiency (e), and 60% energy efficiency (f) for the medium capital cost scenario.

An analysis of six energy efficiency scenarios evaluates the correlation between the decentralization level and ammonia production cost and distribution distance (Figure 7). This analysis covers production and distribution networks between 1 and 6000 global regional production locations. As the decentralization level increases, the ammonia production cost increases and the average distribution distance decreases (Figure 7a). For low-efficiency ‘Black Box’ systems, the average ammonia production cost increases from a minimum of 1270  for 1 production region to a maximum of 1930  for 6000 production regions. Similarly, in medium-efficiency ‘Black Box’ systems, the average ammonia production cost increases from a minimum of 670  for 1 production region to a maximum of 1010  for 6000 production regions. Finally, in high-efficiency ‘Black Box’ systems, the average ammonia production cost increases from a minimum of 465  for 1 production region to a maximum of 700  for 6000 production regions. Decentralization has the opposite effect on the average distribution distance. Interestingly, all of the scenarios have the same trend between the number of production regions and the average distribution distance (Figure 7b). For all scenarios, the average ammonia distribution distance decreases from a maximum of 7627 km for 1 production region to a minimum of 155 km for 6000 production regions.

A Pareto frontier highlights the trade-off between production cost and distribution distance in optimal solutions (Figure 7c). The marginal change in the average ammonia production cost is inversely proportional to the average distribution distance. For instance, decreasing the average distribution distance by 50% from the most centralized scenario (one facility) results in a 5% increase in the average ammonia production costs. In contrast, decreasing the average distribution distance by 75% from the most centralized scenario (one facility) results in a 15% increase in the average ammonia production costs. Finally, decreasing the average distribution distance by 95% from the most centralized scenario (one facility) results in a 35% increase in the average ammonia production costs. These results highlight the trade-off in decentralization and production cost, emphasizing the importance of selecting the number of regional production regions so that the production costs remain low while having low distribution distances that are resilient to changes in transportation costs.

**Figure 8:**
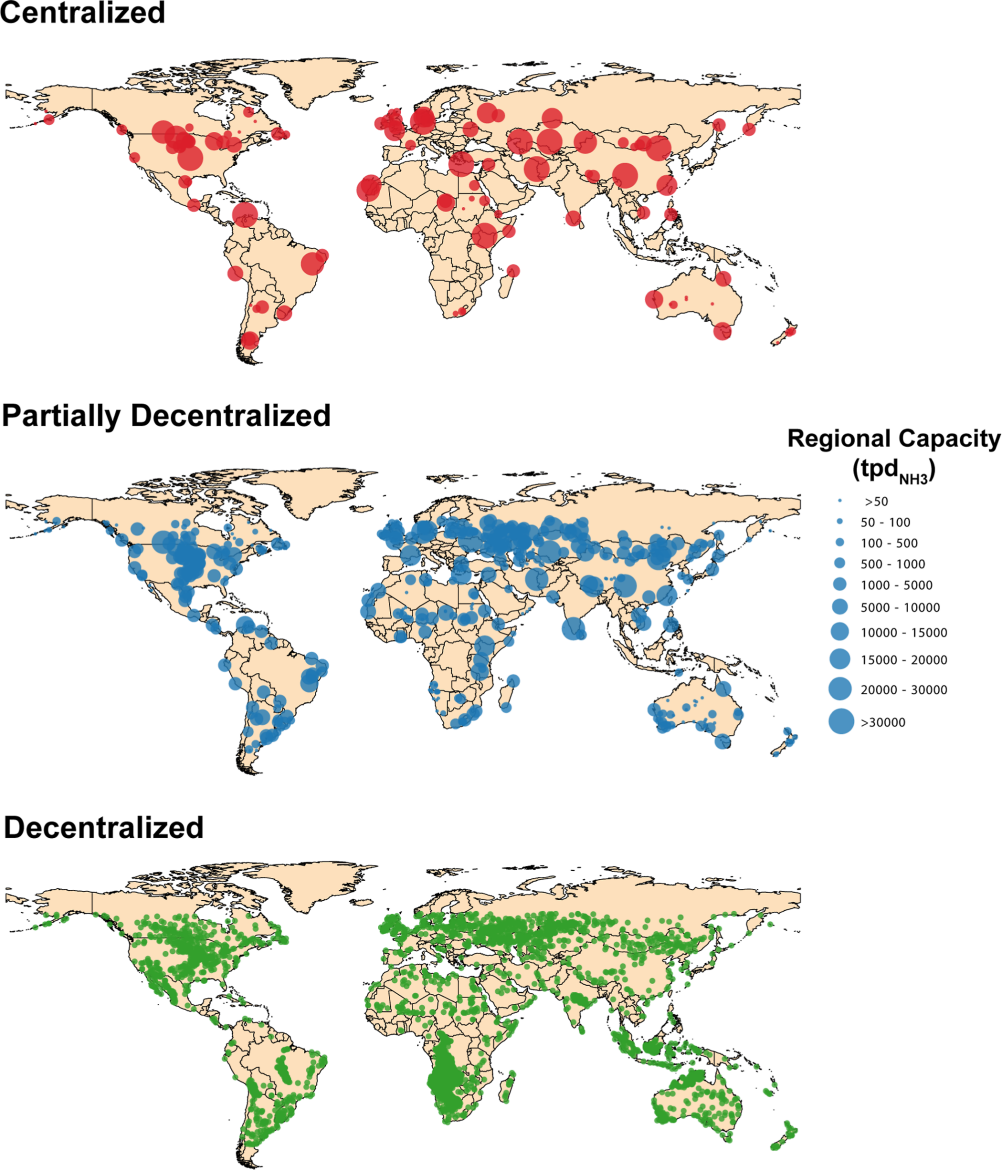
Spatial distribution of ammonia production facilities for a centralized, partially decentralized, and fully decentralized scenario. The scenarios were optimized assuming the medium cost scenario and a 40% energy efficiency. The centralized scenario has a total of 100 production regions. The partially decentralized scenario has a total of 500 production regions and the decentralized scenario has a total of 3000 production regions.

To understand the production cost and the response to changes in the transportation cost of different wind and photovoltaic electricity-driven ammonia production networks, we performed a network optimization analysis for centralized (100 production regions), partially decentralized (500 production regions), and fully decentralized scenarios (3000 production regions). Then, we studied the behavior of each decentralization scenario with respect to changes in transportation costs for each decentralization scenario and energy efficiency scenario (Figure 7d–f). The centralized scenario (100 production regions) resembles the current ammonia production infrastructure through the Haber–Bosch process (Figure 8). In contrast, the proposed centralized scenario for wind and photovoltaic electricity-driven ammonia production shifts production from areas with developed and inexpensive natural gas resources to areas with good wind and solar resources (Figure 8). Consequently, with the implementation of wind and photovoltaic electricity-driven ammonia, a bulk of the production capacity migrates to the global south, which historically has produced low quantities of ammonia.

A partially decentralized scenario (500 production regions) results in production facilities located closer to agricultural centers (Figure 8). The average distance between the production facilities and the agricultural centers decreases from 990 km in the centralized scenario to 510 km. This scenario still places great importance on the production cost over the distribution distance. Finally, a fully decentralized scenario results in smaller facilities near the location of agricultural centers (Figure 8). In this scenario, the average distance between the production sites and agricultural centers is 240 km. This scenario would significantly simplify the global ammonia supply chain by minimizing trade between countries and reducing transportation distances. Interestingly, the fully decentralized scenario exhibits a notable shift in the energy sources driving ammonia production. While the centralized and partially decentralized scenarios are predominantly driven by wind energy, the fully decentralized scenario sees the emergence of more photovoltaic-driven ammonia production locations. This is evident in the identification of new production locations in the southwest United States and western Mexico.

The more decentralized the production infrastructure, the less sensitive it is to uncertainties in transportation costs (Figure 7). For example, doubling the transportation cost for the centralized scenario from 0.09 -km results in a 178  increase in the total ammonia cost. For the partially decentralized scenario, the same change in transport cost results in a 90  increase in the cost of ammonia. For a fully decentralized scenario, the same change in transportation cost results in a 45  increase in ammonia cost. Through this analysis, we can identify the transportation cost that would trigger a move toward decentralization of the wind and photovoltaic electricity-driven ammonia production infrastructure.

- The centralized scenario results in the lowest overall ammonia cost if transportation costs remain under 0.24 -km for low-efficiency systems (Figure 7d), 0.12 -km for medium-efficiency systems (Figure 7e), and 0.09 -km for high-efficiency systems (Figure 7f).
- The partially decentralized scenario results in the lowest overall ammonia cost if the transportation costs are between 0.24 -km and 0.61 -km for low-efficiency systems, between 0.12 -km and 0.32 -km for medium-efficiency systems, and between 0.09 -km and 0.22 -km for high-efficiency systems.
- The fully decentralized scenario results in the lowest overall ammonia cost if the transportation costs are above 0.61 -km for low-efficiency systems, 0.32 -km for medium-efficiency systems, and 0.22 -km for high-efficiency systems.

Our results highlight the benefits of a decarbonized and decentralized ammonia supply chain by showing that the decentralization of wind and photovoltaic electricity-driven ammonia production leads to reduced levels of price sensitivity. With the price of agricultural commodities increasing rapidly, the current system of ammonia production places unnecessary strain on global food security due to its susceptibility to volatility in energy prices.^51^ The ammonia produced by the Haber–Bosch process is highly dependent on natural gas, which accounts for 70–90% of its production costs.^52^ This relationship is asymmetric, with positive changes in energy prices having a stronger and longer-lasting effect on agriculture commodities than a negative change.^53^ Wind and photovoltaic electricity-driven ammonia production would better isolate ammonia and thus food prices from the impacts of volatility in the natural gas market.

We highlight the balance between the low production costs achieved by centralized systems and the short transportation distances attained in a decentralized market. A decentralized wind and photovoltaic electricity-driven ammonia production network has production costs of 143  higher than a centralized production network for systems with 40% energy efficiency. However, a decentralized wind and photovoltaic electricity-driven ammonia production network is three times less sensitive to changes in transportation costs than a centralized network. Policies that address the higher price of decentralized production would facilitate lower prices that maintain greater price stability, potentially proving to be more cost-effective than current policies in place to maintain food price stability.

## Supporting Information Available

Details on the methodology used in the study, data sets used, and tables highlighting the relevant results discussed in the study (PDF)
