# Supplementary material for: Achieving Decentralized, Electrified, and Decarbonized Ammonia Production
Source: Environ Sci Technol. 2024 Apr 11;58(16):6964–77. doi: 10.1021/acs.est.3c10751 (PMC11044596; doi:10.1021/acs.est.3c10751)
Supplement: Supplementary file 1 — es3c10751_si_001.pdf [file es3c10751_si_001.pdf]

Supporting Information: Achieving Decentralized, Electrified, and Decarbonized Ammonia Production

Carlos A. Fernández,[†] Oliver Chapman,[‡] Marilyn A. Brown,[‡] Christian Alvarez Pugliese,[¶] and Marta C. Hatzell[*,†,§]

†*George W. Woodruff School of Mechanical Engineering, Georgia Institute of Technology, Atlanta, Georgia 30318, United States*

‡*School of Public Policy, Georgia Institute of Technology, Atlanta, Georgia 30332, United States*

¶*Department of Chemical Engineering, Texas Tech University, Lubbock, Texas 79409, United States*

§*School of Chemical and Biomolecular Engineering, Georgia Institute of Technology, Atlanta, Georgia 30318, United States*

E-mail: marta.hatzell@me.gatech.edu

Number of pages in supporting information: 58

Number of tables: 32

Number of figures: 7

Contents

1 Variables **S7**

2 Open-Source Code Availability **S12**

3 Input Datasets **S12**

4 Techno-economic model **S14**

4.1 Economic and Technology Parameters and Scenarios S14

5 Electrified Haber-Bosch Process Production Cost Model **S16**

6 'Black Box' Process Production Cost Model **S18**

7 Subsystem Capital Cost Estimation **S20**

7.1 Air Separation Unit System Model and Capital Cost Estimation S20

7.2 PEM Water Electrolysis Model and Capital Cost Estimation S22

7.3 Haber-Bosch Loop System Model and Capital Cost Estimation S23

7.4 Storage Cost Model and Capital Cost Estimation S28

7.5 'Black Box' Ammonia Reactor Model and Capital Cost Estimation S29

7.6 Solar Photovoltaic Cost Model and Capital Cost Estimation S31

7.7 Wind Energy Model and Capital Cost Estimation S33

8 Calculating Fertilizer Demand **S35**

9 Multi-objective Optimization **S37**

10 Methane-Fed Haber-Bosch Process Production Cost Model **S39**

11 Supporting Tables **S46**

12 Supporting Figures S50

List of Figures

1 Methane-fed Haber-Bosch process diagram. S39

2 Normalized capital cost of different technology scenarios with variations in production capacity. S50

3 Average daily global tilted irradiance at the optimal tilt angle obtained from the Global Solar Atlas 2.0, a free, web-based application is developed and operated by the company Solargis s.r.o. on behalf of the World Bank Group, utilizing Solargis data, with funding provided by the Energy Sector Management Assistance Program (ESMAP). For additional information: https://globalsolaratlas.info S51

4 Air Temperature at 2 m above ground level obtained from the Global Wind Atlas 3.0, a free, web-based application developed, owned and operated by the Technical University of Denmark (DTU). The Global Wind Atlas 3.0 is released in partnership with the World Bank Group, utilizing data provided by Vortex, using funding provided by the Energy Sector Management Assistance Program (ESMAP). For additional information: https://globalwindatlas.infoS52

5 Level 1 practical PV potential obtained from the Global Solar Atlas 2.0, a free, web-based application is developed and operated by the company Solargis s.r.o. on behalf of the World Bank Group, utilizing Solargis data, with funding provided by the Energy Sector Management Assistance Program (ESMAP). For additional information: https://globalsolaratlas.info S53

6 Locations of global agriculture centers.[1] S54

7 Water Stress Score. Source: WRI Aqueduct. Source: aqueduct.wri.org. . . S55

List of Tables

S2 Geospatial Input Datasets S13
S3 Techno-economic parameters used in the sensitivity analysis and capital cost scenarios. S14
S4 Adsorber Parameters.[2] S20
S5 Pressure Swing Adsorption System Sizing for 1,000 tpd_{NH3} capacity. S20
S6 Cost Scaling Parameters for PSA.[3] S22
S7 Installation Factors for PSA.[3] S22
S8 PEM Electrolyzer Parameters.[4] S23
S9 Thermodynamic and kinetic parameters for the LHHW model for ammonia production rate in wustite.[5] S24
S10 Pressure Swing Adsorption System Sizing for 1,000 tpd_{NH3} capacity from ASPEN Plus model. S25
S11 Cost Scaling Parameters for the Haber-Bosch loop.[3] S26
S12 Installation Factors for the Haber-Bosch loop.[3] S27
S13 Electrified Haber-Bosch Energy Expenditure S27
S14 Electrified Haber-Bosch Material Requirement S28
S15 Electrified Haber-Bosch storage sizing parameters approximated from Bose et al.[6] S28
S16 'Black Box' System Energy Expenditure S30
S17 Material flow for the methane-fed Haber-Bosch process S40
S18 Methane-fed Haber-Bosch System Sizing for 1,350 tpd_{NH3} capacity from ASPEN Plus model. S42
S19 Methane-fed Haber-Bosch System Sizing for 1,350 tpd_{NH3} capacity from ASPEN Plus model (cont.). S43
S20 Cost Scaling Parameters for the methane-fed Haber-Bosch.[3] S43

S21 Installation Factors for the methane-fed Haber-Bosch.[3] S44
S22 Results from Figure 2. Average average geographic cost of ammonia production. S46
S23 Results from Figure 2. Natural gas prices and economic viability. S46
S24 Results from Figure 3. The optimal number of regional production locations. S47
S25 Results from Figure 3. Optimal ammonia production cost. S47
S26 Results from Figure 3. Optimal ammonia distribution cost. S47
S27 Results from Figure 3. Optimal ammonia distribution distance. S47
S28 Results from Figure 4a. Effect of discount rate on the ammonia cost. S48
S29 Results from Figure 4b. Effect of discount rate on the average distribution distance. S48
S30 Results from Figure 4c. Effect of discount rate on the number of production regions. S48
S31 Results from Figure 4d. Effect of discount rate on the average production capacity. S48
S32 Results from Figure 5. Wind and photovoltaic electricity-driven ammonia and water uncertainty. S49

1 Variables

Variable	Description	Units	Equation
α_p	Temperature Coefficient	$\%$	35
Δdeg	Grid Resolution	$degrees$	43, 44
η_{NH3}	Ammonia Energy Efficiency	$\%$	27
η_o	Nominal Solar Panel Efficiency	$\%$	35
η_{PV}	Solar Panel Efficiency	$\%$	32, 35, 36
ρ	Density	$\frac{kg}{m^3}$	10, 19, 49
A	Grid Area	km^2	45
a, b, n	Cost Scaling Parameters	$--$	11 20, 50
A_{PV}	PV Area	m^2	32, 36
A_z, B_z, C_z, D_z	LHHW Parameters	$--$	17
C	Uninstalled Capital Cost	USD	11, 20, 21<br>50, 51
$CapEx_{Battery}$	Battery Capital Cost	USD	23, 25
$CapEx_{BB}$	'Black Box' Capital Cost	USD	29, 5
$CapEx_{eBB}$	'Black Box' System Capital Cost	USD	5, 7
$CapEx_{eHB}$	Electrified Haber-Bosch Capital Cost	USD	1, 3
$CapEx_{H2_{Storage}}$	H2 Storage Capital Cost	USD	23, 24
$CapEx_{HB}$	HB Capital Cost	USD	22, 1
$CapEx_{mHB}$	Methane-Fed Haber-Bosch Capital Cost	USD	52, 53
$CapEx_{N2_{Storage}}$	N2 Storage Capital Cost	USD	23, 24
$CapEx_{NH3_{Storage}}$	NH3 Storage Capital Cost	USD	23, 26, 6
$CapEx_{PEM}$	PEM Capital Cost	USD	14, 15, 1
$CapEx_{PSA}$	PSA Capital Cost	USD	13, 1, 5

$CapEx_{PV}$	PV Capital Cost	USD	37, 1, 5<br>6
$CapEx_{Separations}$	Separations Capital Cost	USD	29
$CapEx_{Storage}$	Storage Capital Cost	USD	23, 1
$CapEx_{Wind}$	Wind Capital Cost	USD	41, 1, 5
C_{BB}	'Black Box' Power Scaling Factor	$\frac{USD}{kW}$	29
C_i	Installed Capital Cost	USD	12, 13, 21<br>51, 52
C_{PEM}	PEM Power Scaling Factor	$\frac{USD}{kW}$	14
C_{PV}	PV Capital Cost Factor	$\frac{USD}{kW}$	37
CF	Wind Capacity Factor	$\frac{kW_{mean}}{kWp}$	40
C_t	Transportation Cost	$\frac{USD}{ton_{NH3}-km}$	46
C_{Wind}	Wind Capital Cost Factor	$\frac{USD}{kW}$	41
d	Discount Rate	$\%$	3, 7, 53
D_c	Column Diameter	m	9, 10, 18<br>19, 48, 49
deg	Yearly Degradation Rate	$\%$	4, 8
d_t	Distribution Distance	km	46, 47
Ea	Activation Energy	$\frac{J}{mol_{NH3}}$	16
E_{NH3}	Ammonia Energy Consumption	$\frac{kWh}{kg_{NH3}}$	27, 28
E_{PSA}	PSA Energy Consumption	$\frac{kWh}{kg_{NH3}}$	
$E_{Required}$	System's Energy Consumption	$\frac{kWh}{yr}$	31, 32, 39<br>40, 42
E_{Total}	'Black Box' Energy Consumption	$\frac{kWh}{kg_{NH3}}$	28, 31, 39
f_a	Fugacity	$\frac{N}{m^2}$	16
$f_{Capacity}$	Capacity Cost Factor	$\frac{USD}{MWh}$	25

$f_{H2/N2}$	H2/N2 Storage Cost Factor	$\frac{USD}{ton_{NH3}}$	24
f_i	Installation Factor	$--$	12, 21, 51
f_{NH3}	NH3 Storage Cost Factor	$\frac{USD}{ton_{NH3}}$	26
f_{Power}	Power Cost Factor	$\frac{USD}{MW}$	25
$frac_{NH3}$	NH3 Storage Fraction	$--$	26
GCR	Ground Coverage Ratio	$--$	32, 36
GTI	Global Tilted Irradiance	$\frac{kWh}{m^2-day}$	32, 33
h	Pixel Height	km	44, 45
h_o	Length of a Degree Latitude at the Equator	km	44
k	Kinetic Factor	$\frac{kmol_{NH3}}{kg_{catalyst}}$	16
kW_{DC}	Wind/Solar Rated Power	kW	28, 36, 37<br>38, 40, 41<br>42
$K_{Forward}$	Forward Equilibrium Constant	$--$	16, 17
K_i	Adsorption Equilibrium Constant	$--$	16
$K_{Reverse}$	Reverse Equilibrium Constant	$--$	16, 17
lat	Latitude	$degrees$	43
lat_1, lon_1	Production Location Coordinates	$degrees$	47
lat_2, lon_2	Farm Location Coordinates	$degrees$	47
L_c	Column Length	m	10, 19, 49
$LCOA_{NH3}$	Levelized Cost of Ammonia	$\frac{USD}{ton_{NH3}}$	3, 7, 46<br>53
MM_{NH3}	Ammonia Molar Mass	$\frac{g}{mol}$	27
M_{NH3}	Nameplate Yearly Ammonia Production	$\frac{kg}{yr}$	31, 39, 39<br>26, 4, 8<br>53

$NH3_t$	Yearly Ammonia Production	$\frac{ton_{NH3}}{year}$	3, 4, 7<br>8
$NOCT$	Nominal Operating Cell Temperature	$°C$	34
$OpEx_{BB}$	'Black Box' O&M Cost	USD	30
$OpEx_{eBB}$	'Black Box' System O&M Cost	USD	6, 7
$OpEx_{eHB}$	Electrified Haber-Bosch O&M Cost	USD	2, 3
$OpEx_{HB}$	HB O&M Cost	USD	2
$OpEx_{mHB}$	Methane-Fed Haber-Bosch O&M Cost	USD	53
$OpEx_{PEM}$	PEM O&M Cost	USD	15, 2
$OpEx_{PSA}$	PSA O&M Cost	USD	2
$OpEx_{PV}$	PV O&M Cost	USD	38, 2
$OpEx_{Wind}$	Wind O&M Cost	USD	42, 2, 6
$O\&M_{BB}$	'Black Box' O&M Factor	$--$	30
$O\&M_{PEM}$	PEM O&M Factor	$--$	15
$O\&M_{PV}$	PV O&M Factor	$\frac{USD}{kW}$	38
$O\&M_{Var}$	Wind Variable O&M Factor	$\frac{USD}{MWh}$	42
$O\&M_{Wind}$	Wind O&M Factor	$\frac{USD}{kW}$	42
$P_{Battery}$	Battery Power	MW	25
P_{BB}	'Black Box' Rated Power	kW	28, 29
P_i	Design Pressure	$\frac{N}{m^2}$	9, 18, 48
P_{PEM}	PEM Peak Power	kW	14
PV_{out}	Photovoltaic Power Potential	$\frac{kWh}{kWp}$	33
R	Universal Gas Constant	$\frac{kJ}{kmol-K}$	16
R_{Earth}	Radius of Earth	km	47
r_{NH3}	Reaction Rate	$\frac{kmol_{NH3}}{kg_{catalyst}}$	16
S	Maximum Solar Irradiance	$\frac{kW}{m^2}$	33, 34, 36

$S_{Battery}$	Battery Capacity Factor	$\frac{MWh}{ton_{NH3}}$	25
$Score$	Optimization Score	$--$	46
SE	Maximum Allowable Stress	$\frac{N}{m^2}$	9, 18, 48
$S_{H2/N2}$	H2/N2 Storage Size Factor	$\frac{ton_{H2/N2}}{ton_{NH3}}$	24
SM	Shell Mass	kg	10, 19, 49
T	Reaction Temperature	K	16, 17
t	Year in Lifetime	$year$	3, 4, 7<br>8, 53
T_{air}	Air Temperature	$^{\circ}C$	34
T_{Cell}	PV Module Temperature	$^{\circ}C$	34, 35
tpd_{NH3}	Ammonia Production Capacity	$\frac{ton_{NH3}}{day}$	24, 25
t_w	Wall Thickness	m	9, 10, 18<br>19, 48, 49
w	Pixel Width	km	43, 45
w_1, w_2	Optimization Weights	$--$	46
WS	Water Stress	–	46
w_o	Length of a Degree Longitude at the Equator	km	43

2 Open-Source Code Availability

The code, models, data, and results included in this paper are now available open source on GitHub. This repository contains the models discussed in our paper, enabling researchers to reproduce our results and further explore the methodologies presented.

The GitHub repository can be accessed at github.com/caturof96/SusNH3Prod. We encourage readers to explore the codebase, provide feedback, and utilize it for their research endeavors. We believe that making our code openly accessible contributes to the transparency and reproducibility of our work, fostering collaboration and advancing scientific knowledge in the field.

3 Input Datasets

In order to calculate the geospatial distribution of ammonia production costs, we used several geospatial datasets to determine the resource availability for solar energy, wind energy, water resources, and agricultural resources. Table S2 outlines the datasets we used and the source for each dataset. The first dataset describes the geospatial distribution of the Global Tilted Irradiance (GTI) at the optimal tilt angle (Fig. 3). This dataset represents the daily solar input per unit of area in the solar panel ($kWh/m^2/day$). The second dataset describes the air temperature surrounding the solar panel and it is used to calculate the efficiency of the solar panel when compared to the nominal efficiency (Fig. 4). The third dataset is the solar power potential (kWh/kWp), which is used to size the solar PV system and the ammonia production system (Fig. 5). This dataset excludes areas that are unsuitable for the implementation of PV-driven ammonia such as areas with rugged terrain, urbanized or industrial areas, forests, and areas that are too distant from population centers.[7] The fourth dataset describes the capacity factor for wind power. The capacity factor represents the fraction of the rated power at which the wind turbine

operates on average at any location in a year. Finally, the last dataset describes the water stress projections for 2050. Water stress is defined as the ratio of water usage over available water.

Table S2: Geospatial Input Datasets

**Dataset Name**	**Abbreviation**	**Units**	**Reference**
Globat Tilted Irradiance	GTI	$kWh/m^2/day$	[7]
Air Temperature at 2 m above ground level	T_{air}	$^{\circ}C$	[7]
Photovoltaic Power Potential	PV_{out}	kWh/kWp	[7]
Wind Capacity Factor	CF	kWm/kWp	[8]
Water Stress	WS	–	[9]

The datasets for the global tilted irradiance, air temperature, and photovoltaic power potential are used to calculate the ammonia production cost from solar photovoltaic power. The dataset for the wind capacity factor is used to calculate the ammonia production cost from wind power. Finally, the water stress data is used to estimate the possible water stress at the suggested production locations. We used baseline water stress projections for 2050 to ensure that our results are accounting for changes in water availability due to climate change. However, we find that the suggested areas are similar when using water stress projections for 2050 and current water stress values.

4 Techno-economic model

In order to calculate the ammonia production cost from an electrified Haber-Bosch process and a 'Black Box' electrified process, we used techno-economic models based on the production capacity for an ammonia production facility (M_{NH3}).

4.1 Economic and Technology Parameters and Scenarios

The economic and technology parameters used for calculating the ammonia production costs are outlined in Table S3. These parameters are used for the sensitivity analysis and the different cost scenarios. The capital cost scenarios include variations in PV, wind, and electrolyzer capital costs, while the energy efficiency scenarios only include variations in the system's energy efficiency.

Table S3: Techno-economic parameters used in the sensitivity analysis and capital cost scenarios.

**Parameter**	**Optimistic**	**Baseline**	**Pesimistic**
PV Overnight Capital Cost (C_{PV} – USD/kW)[10]	460	767	1,323
PV $O\&M$ ($O\&M_{PV}$ – USD/kW-yr)[11]	7.56	23.525	39.49
PV Lifetime (n_{PV} – years)[11]	30	35	40
PV Degradation (deg_{PV})[12]	0.2	0.5	0.8
Wind Overnight Capital Cost (C_{Wind} – USD/kW)[10]	676	1,127	1,412
Wind $O\&M$ ($O\&M_{Wind}$ – USD/kW-yr)[11]	7.56	23.525	39.49
Wind Var. $O\&M$ ($O\&M_{Var}$ – USD/MWh)[11]	4.82	8.81	23
Wind Lifetime (n_{Wind} – years)[11]	15	17.5	20
Wind Degradation (deg_{Wind})[13]	1.2	1.6	2
Discount Rate (d)	3	7	10
Energy Efficiency (η_{NH3})	20	40	60
PEM Overnight Capital Cost (C_{PEM} – USD/kW)[4]	200	550	900
PEM $O\&M$ ($O\&M_{PEM}$ – %)[6,14]	2	5	10
Ammonia Storage Fraction (r_{NH3})	0.1	0.25	1
Ammonia Storage Cost (f_{NH3} – USD/ton)[15]	500	810	1,000

When modeling the capital cost of the electrified Haber-Bosch process and the electrochemical 'Black Box' system we take into consideration the scale at which the system

operates. We size the electrolyzers with linear scaling factors and all other components with non-linear scaling factors (Figure S2). We show that the scaling factor for the electrified Haber-Bosch model is 0.835. In contrast, for the Black-Box model, the scaling factor is 0.985. A scaling factor closer to 1 shows linearity in the scalability of the Black Box systems.

5 Electrified Haber-Bosch Process Production Cost Model

We calculate the ammonia production cost for the electrified Haber-Bosch process with a set production capacity (tpd_{NH3}). First, we calculate the average nitrogen and hydrogen needs using the values in Table S14. These values are used to convert ammonia flowrate to nitrogen (tpd_{N2}) and hydrogen (tpd_{H2}) feedstock flowrates. The average ammonia and nitrogen flowrates are used to calculate the Haber-Bosch loop and Pressure swing adsorption capital costs, respectively. This is because these systems are assumed to operate consistently throughout the year. As such they are sized to operate at high capacity factors. The wind and solar systems were sized to produce the total amount of energy consumed by the system yearly to produce the desired amount of ammonia. However, the wind and solar systems operate intermittently. As such, the hydrogen electrolyzer was sized to operate intermittently and adjust to the intermittency of solar and wind systems. The hydrogen electrolyzer was sized by taking the peak power from the wind and PV systems and subtracting the power needed for the Haber-Bosch loop and PSA systems. The total capital cost for an electrified Haber-Bosch process is calculated using equation 1.

$$CapEx_{eHB} = CapEx_{Wind/PV} + CapEx_{HB} + CapEx_{PEM} + CapEx_{PSA} + CapEx_{Storage} \quad (1)$$

Where the capital cost of the Wind and PV electricity systems were calculated using equations 31-41, the capital cost for the Haber-Bosch loop was calculated using equations 16-22, the capital cost for the PSA system was calculated using equation 9-13, the capital cost for the PEM electrolyzer was calculated using equation 14, and the storage capital costs were calculated using equations 23-26. Similarly, the total operation and maintenance cost for an electrified Haber-Bosch process is calculated using equation 2.

$$OpEx_{eHB} = OpEx_{Wind/PV} + OpEx_{HB} + OpEx_{PEM} + OpEx_{PSA} \tag{2}$$

The ammonia production cost – or levelized cost of ammonia – is a function of the discounted sum of the yearly costs over the discounted sum of the yearly ammonia produced for the entire lifetime of the project. The ammonia production cost – or levelized cost of ammonia – can be calculated using equation 3.

$$LCOA_{NH3} = \frac{CapEx_{eHB} + \sum_{t=0}^{t=lifetime} \frac{OpEx_{eHB,t}}{(1+d)^t}}{\sum_{t=0}^{t=lifetime} \frac{NH3_t}{(1+d)^t}} \tag{3}$$

Where CapEx is the initial capital investment, OpEx is the yearly operation costs, d is the discount rate, t is the year, and NH3$_t$ is the yearly ammonia production. The yearly ammonia production NH3$_t$ at any year (t) can be calculated using equation 4

$$NH3_t = M_{NH3} * (1 - deg * t) \tag{4}$$

Where M_{NH3} is the nameplate ammonia capacity, deg is the yearly degradation rate in Table S3, meaning that the production capacity decreases by that rate every year from the initial capacity.[16]

6 'Black Box' Process Production Cost Model

The electrochemical 'Black Box' process is assumed to operate intermittently following the wind and solar profiles. As such, the wind and solar systems are sized to produce the yearly energy needed to produce the average ammonia capacity in a year. However, the ammonia, PSA, and separation systems are sized to operate at the peak production capacity following the wind and solar energy profiles. The capital cost for the electrochemical 'Black Box' process can be calculated using equation 5.

$$CapEx_{eBB} = CapEx_{Wind/PV} + CapEx_{BB} + CapEx_{PSA} \tag{5}$$

Where the capital cost of the Wind and PV electricity systems were calculated using equations 31-41, the capital cost for the 'Black Box' ammonia production system was calculated using equations 27-29, the capital cost for the PSA system was calculated using equation 9-13. Similarly, the total operation and maintenance cost for an electrified Haber-Bosch process is calculated using equation 6.

$$OpEx_{eBB} = OpEx_{Wind/PV} + OpEx_{BB} + OpEx_{PSA} + CapEx_{NH3_{Storage}} \tag{6}$$

The ammonia production cost – or levelized cost of ammonia – is a function of the discounted sum of the yearly costs over the discounted sum of the yearly ammonia produced for the entire lifetime of the project. The ammonia production cost – or levelized cost of ammonia – can be calculated using equation 7.

$$LCOA_{NH3} = \frac{CapEx_{eBB} + \sum_{t=0}^{t=lifetime} \frac{OpEx_{eBB,t}}{(1+d)^t}}{\sum_{t=0}^{t=lifetime} \frac{NH3_t}{(1+d)^t}} \tag{7}$$

Where CapEx is the initial capital investment, OpEx is the yearly operation costs, d is the discount rate, t is the year, and $NH3_t$ is the yearly ammonia production. The yearly ammonia production $NH3_t$ at any year (t) can be calculated using equation 8

$$NH3_t = M_{NH3} * (1 - deg * t) \tag{8}$$

Where M_{NH3} is the nameplate ammonia capacity, deg is the yearly degradation rate in Table S3, meaning that the production capacity decreases by that rate every year from the initial capacity.[16]

7 Subsystem Capital Cost Estimation

7.1 Air Separation Unit System Model and Capital Cost Estimation

The capital cost and energy expenditure of a pressure swing adsorption (PSA) system are calculated based on the nitrogen output (tpd_{N2}). The pressure swing adsorption system consists of two interconnected adsorber columns and a compressor. The parameters for the adsorber are based on experimental results and are shown in Table S4. The production capacity for the PSA system is calculated using the factor in table S14 for the electrified Haber-Bosch process and stoichiometrically for the 'Black Box' Model.

Table S4: Adsorber Parameters.[2]

Parameter	Abbreviation	Value	Units
Adsorber Pressure	P_{PSA}	6	bar
Adsorber Temperature	T_{PSA}	25	$^{\circ}C$
Compressor Efficiency	η_{Comp}	0.75	%
Catalyst Capacity	$R_{Catalyst}$	68	$\frac{NCMH}{m^3}$
Air Demand	AD	3.27	$\frac{NCMH_{air}}{NCMH_{N2}}$
Inter-particle voidage	ϵ_P	0.404	$\frac{m^3_{void}}{m^3_{bed}}$

The ASPEN simulation is used to estimate the energy expenditure for the PSA system and appropriately size each component. Table S5 shows the component sizing and energy expenditure for a PSA system with a capacity of 1,000 tons of nitrogen per day.

Table S5: Pressure Swing Adsorption System Sizing for 1,000 tpd_{NH3} capacity.

Component	Size	Units
Compressor Power	11,176	kW
Catalyst Volume	490	m^3
Adsorber Bed Volume (each)	411	m^3
Aspect Ratio (L/D)	10	–

These components are scaled based on the adjusted values from Table S5 for systems with other production capacities. The energy expenditure for the PSA system based on

the ASPEN simulation is 0.27 kWh/kg_{N2}, which agrees well with the values found in the literature.[6] The catalyst price (carbon molecular sieves) was assumed to be 5,500 USD/m^3 (Alibaba), the length of the column is assumed to be 10 times the diameter of the column, and the design pressure is assumed to be 10% higher than the operating pressure. The wall thickness (t_w) of the adsorber bed was calculated from the diameter and the design pressure (equation 9).[3]

$$t_w = \frac{P_i * D_c}{2 * SE - 1.2 * P_i} \tag{9}$$

where P_i is the design pressure for the adsorber column (which is 10% higher than the operating pressure in N/m^2), D_c is the column diameter in meters, and SE is the maximum allowable stress for 304 stainless steel at 25$^{\circ}C$ (20 ksi or 1.38e8 N/m^2). From the wall thickness, length, and diameter, we can calculate the adsorber column shell mass using equation 10.[3]

$$SM = \pi * D_c * L_c * t_w * \rho \tag{10}$$

where D_c is the column diameter in meters, L_c is the column length in meters, t_w is the column wall thickness in meters, and ρ is the density for 304 stainless steel (ρ = 8,000 kg/m^3). The compressor power and the absorbers' shell mass are used to calculate the uninstalled capital cost for the PSA system in Equation 11.[3]

$$C = a + b * S^n \tag{11}$$

The values for each one of the components of this equation for the pressure swing adsorption system are highlighted in Table S6.

The installed capital cost for each parameter is calculated by multiplying the uninstalled capital cost by the installation factor (Equation 12 and Table S7).[3]

Table S6: Cost Scaling Parameters for PSA.[3]

Equipment	Units for S	a	b	n
Centrifugal Compressor	driver power (kW)	580,000	20,000	0.6
Vertical Pressure Vessel (304 ss)	shell mass (kg)	17,400	79	0.85

$$C_i = f_i * C \tag{12}$$

Table S7: Installation Factors for PSA.[3]

Equipment	Installation Factor
Centrifugal Compressor	2.5
Vertical Pressure Vessel (304 ss)	4

The installed capital cost represents the inside battery limit (ISBL) costs. Additional costs, referred to as the outside battery limit (OSBL) costs are assumed to be 30% of the ISBL costs. Additional design and engineering costs are 30% of the total plant costs (ISBL + OSBL) and contingency costs are 10% of the total plant costs (ISBL + OSBL).[3] As such, the total capital costs related to the PSA system are 1.82 times the ISBL costs after adding OSBL, design and engineering, and contingency (equation 13).

$$CapEx_{PSA} = 1.82 * C_i \tag{13}$$

The yearly operation and maintenance costs for the PSA system ($OpEx_{PSA}$) are 5% of the capital cost.

7.2 PEM Water Electrolysis Model and Capital Cost Estimation

The PEM water electrolyzer is integrated with the main Haber-Bosch loop in order to heat up the feed water to the operating temperature. The parameters for the PEM water electrolyzer model are outlined in Table S8. The hydrogen electrolyzer was sized by

taking the peak power coming from the wind and PV systems and substracting the power needed for the Haber-Bosch loop and PSA systems.

Table S8: PEM Electrolyzer Parameters.[4]

Parameter	Abbreviation	Value	Units
PEM Pressure	P_{PEM}	6	bar
PEM Temperature	T_{PEM}	80	$^{\circ}C$
PEM Energy Expenditure	E_{PEM}	45	kWh/kg_{H2}
PEM Intalled CapEx	C_{PEM}	200-900	$\frac{USD}{kW}$

The total capital costs related to the Hydrogen Electrolyzer system were calculated using a single power-scaling parameter (equation 14). This parameter is assumed to include the installation, offsite, design and engineering, and contingency cost due to the uncertainty of the technology's cost in the future and the fact that we are varying the parameter and studying the sensitivity of the parameter throughout the paper.

$$CapEx_{PEM} = C_{PEM} * P_{PEM} \tag{14}$$

The operation and maintenance cost of the PEM electrolyzer depends on the capital cost of the electrolyzer and the PEM operation and maintenance factor ($OpEx_{PEM}$).

$$OpEx_{PEM} = O\&M_{PEM} * CapEx_{PEM} \tag{15}$$

7.3 Haber-Bosch Loop System Model and Capital Cost Estimation

The capital cost and energy expenditure of a Haber-Bosch ammonia production system (HB) are calculated based on the ammonia production capacity. The system consists of a feed compressor, a recycle compressor, four Haber-Bosch reactors, heat exchangers to control the feed temperature for each reactor and an ammonia separation flash unit. The Haber-Bosch reactors are modeled based using the Langmuir-Hinshelwood-Hougen-Watson (LHHW) equation for wustite catalyst. The reaction rate was calculated using

equation 16.[5]

$$r_{NH3} = (k * e^{\frac{E_a}{RT}})\frac{K_{Forward}\Pi_r f_r^{v_r} - K_{Reverse}\Pi_p f_p^{v_p}}{(\sum_i K_i \Pi_i f_a^{v_i})^n} \quad (16)$$

This equation describes the reaction rate (r in $kmol_{NH3}/kg_{catalyst}$) where k is the kinetic factor ($kmol_{NH3}/kg_{catalyst}$), E is the activation energy (J/mol_{NH3}), T is the reaction temperature (K), R is the universal gas constant (8.314 kJ/kmolK), $K_{Forward}$ is the forward equilibrium constant, $K_{Reverse}$ is the reverse equilibrium constant, K_i is the adsorption equilibrium constant, and f is the fugacity of a component v_x. For the wustite material, the adsorption constant does not exist and the whole adsorption term is canceled with the exponent n = 0. The forward and reverse equilibrium constants are calculated using equation 17.

$$ln(K_z) = A_z + B_z/T + C_z ln(T) + D_z T \quad (17)$$

The parameters used for the LHHW model are outlined in Table S9. These parameters are used to calculate the ammonia production rate in each reactor per mass of catalyst.

Table S9: Thermodynamic and kinetic parameters for the LHHW model for ammonia production rate in wustite.[5]

**Parameter**				
**Stoichiometry**	N_2	H_2	NH_3	
	-0.5	-1.5	1	
**Kinetic constant**	E_a $(kcal/mol)$	k_o $(kmol/(s*kg_{cat})$		
	45	7.47*10^8		
**Rate Expresion**				
**Exponents**	N_2	H_2	NH_3	
**Forward term (v_r)**	1	2.25	-1.5	
**Reverse term (v_p)**	0	-0.75	0.5	
**Coefficients**	A	B	C	D
**Term 1 (K$_{Forward}$)**	-7.8	9218	-5.42	7.8*10^{-4}
**Term 2 (K$_{Reverse}$)**	2.88	0	0	0

The system consists of four Haber-Bosch reactors, one flash separation unit, and a feed compressor to pressurize the inlet nitrogen and hydrogen. The temperature and pressure of each reactor are controlled to balance reaction kinetics (improved kinetics at higher temperatures) and thermodynamic equilibrium (which favors ammonia production at lower temperatures). The temperature and pressure for each reactor are outlined in Table S10.

After the four Haber-Bosch reactors, the ammonia is cooled down to 15 $^{\circ}C$, and the liquified ammonia is separated from the product stream using a flash reactor. The component sizing and energy expenditure for a system producing 1,000 tons of ammonia per day are outlined in Table S10.

Table S10: Pressure Swing Adsorption System Sizing for 1,000 tpd_{NH3} capacity from ASPEN Plus model.

**Component**	**Size**				
Feed Compressor Power (kW)	33,248				
Recycle Compressor Power (kW)	232				
Flash Drum Volume (m^3)	36.3				
Flash Drum Aspect Ratio (L/D)	3	–			
Flash Drum Pressure (bar)	286	–			
Flash Drum Temperature ($^{\circ}C$)	15	–			
Haber-Bosch Reactors	#1	#2	#3	#4	
Inlet Pressure (bar)	292	290	288	286	
Inlet Temperature ($^{\circ}C$)	445	460	410	345	
Catalyst Mass (kg)	8,902	9,712	13,353	29,135	
Reactor Volume (m^3)	2.74	2.99	4.11	8.96	
Reactor Aspect Ratio (L/D)	2	2	2	2	
Heat Exchangers	#1	#2	#3	#4	#5
Area (m^2)	113	71	93	458	117

Additionally, the utilities required in this system are 33,550 kW of electricity and 1.54 MMgal/h of cooling water for a 1,000-ton-per-day output of ammonia. These components are scaled based on the adjusted values from Table S10. The catalyst price for wusite was assumed to be 2 USD/kg and the design pressure for all pressurized reactors was

assumed to be 10% higher than the operating pressure. The wall thickness (t_w) of the Haber-Bosch reactors and flash drum were calculated from the diameter and the design pressure (18).

$$t_w = \frac{P_i * D_c}{2 * SE - 1.2 * P_i} \tag{18}$$

where P_i is the design pressure (which is 10% higher than the operating pressure in N/m^2), D_c is the column diameter in meters, and SE is the maximum allowable stress for 304 stainless steel at 900$^\circ F$ (10.8 ksi or 7.45e7 N/m^2). From the wall thickness, length, and diameter, we can calculate the reactor shell mass using equation 19.

$$SM = \pi * D_c * L_c * t_w * \rho \tag{19}$$

where D_c is the diameter in meters, L_c is the length in meters, t_w is the wall thickness in meters, and ρ is the density for 304 stainless steel (ρ = 8,000 kg/m^3). The compressor power, heat exchanger area, and the absorbers' shell mass are used to calculate the uninstalled capital cost for the HB system in equations 20.

$$C = a + b * S^n \tag{20}$$

The values for each one of the components of this equation for the Haber-Bosch synthesis system are highlighted in Table S11.

Table S11: Cost Scaling Parameters for the Haber-Bosch loop.[3]

Equipment	Units for S	a	b	n
Centrifugal Compressor	driver power (kW)	580,000	20,000	0.6
U-tube shell and tube Heat Exchanger	area (m^2)	28,000	54	1.2
Vertical Pressure Vessel (304 ss)	shell mass (kg)	17,400	79	0.85

The installed capital cost for each parameter is calculated by multiplying the uninstalled capital cost by the installation factor (Equation 21 and Table S12).

$$C_i = f_i * C \tag{21}$$

Table S12: Installation Factors for the Haber-Bosch loop.[3]

Equipment	Installation Factor
Centrifugal Compressor	2.5
Vertical Pressure Vessel (304 ss)	4
Heat Exchanger	3.5

The installed capital cost represents the inside battery limit (ISBL) costs. Additional costs, referred to as the outside battery limit (OSBL) costs are assumed to be 30% of the ISBL costs. Additional design and engineering costs are 30% of the total plant costs (ISBL + OSBL) and contingency costs are 10% of the total plant costs (ISBL + OSBL). As such, the total capital costs related to the PSA system are 1.82 times the ISBL costs after adding OSBL, design and engineering, and contingency (equation 22).

$$CapEx_{HB} = 1.82 * C_i \tag{22}$$

Additionally, the energy expenditure calculated from the ASPEN Plus models for the complete electrified Haber-Bosch process are outlined in Table S13.

Table S13: Electrified Haber-Bosch Energy Expenditure

Parameter	Abbreviation	Value	Normalized Value
PSA	E_{PSA}	0.27 kWh/kg_{N2}	0.224 kWh/kg_{NH3}
PEM	E_{PEM}	45 kWh/kg_{H2}	8.06 kWh/kg_{NH3}
HB	E_{HB}	–	0.8052 kWh/kg_{NH3}
Total	E_{Total}	–	9.09 kWh/kg_{NH3}

Finally, the hydrogen and nitrogen use calculated from the ASPEN Plus models for the complete electrified Haber-Bosch process are outlined in Table S14.

Table S14: Electrified Haber-Bosch Material Requirement

Component	Value	Units
Hydrogen	0.18	kg_{H2}/kg_{NH3}
Nitrogen	0.83	kg_{H2}/kg_{NH3}

The yearly operation and maintenance costs for the HB system ($OpEx_{HB}$) are 5% of the capital cost.

7.4 Storage Cost Model and Capital Cost Estimation

The total storage costs for the electrified Haber-Bosch systems include hydrogen storage, nitrogen storage, battery storage, and ammonia seasonal storage (equation 23).

$$CapEx_{Storage} = CapEx_{H2_{Storage}} + CapEx_{N2_{Storage}} + CapEx_{Battery} + CapEx_{NH3_{Storage}} \quad (23)$$

The capital cost for hydrogen, nitrogen, and battery storage is approximated by calculating the maximum amount of stored hydrogen, nitrogen, and energy stored at any point and adjusting by the cost factors for storing each component using equation X and the values in table S15.

Table S15: Electrified Haber-Bosch storage sizing parameters approximated from Bose et al.[6]

Parameter	Optimistic	Baseline	Pesimistic
H2 Storage (ton_{H2}/tpd_{NH3})	0.48	0.55	0.64
N2 Storage (ton_{N2}/tpd_{NH3})	10	13	16
Battery Storage (MWh/tpd_{NH3})	2.2	2.3	2.4

The capital cost for hydrogen and nitrogen was calculated by multiplying the sizing parameters from table S15 and the storage cost factor using equation 24.

$$CapEx_{H2/N2} = f_{H2/N2} * S_{H2/N2} * tpd_{NH3} \tag{24}$$

where $S_{H2/N2}$ are the storage size factors taken from table S15 and $f_{H2/N2}$ are the storage cost factors for hydrogen (800 USD/kg) and nitrogen (20 USD/kg).[17] The cost for battery storage was calculated assuming a capacity cost of 116,000 USD/MWh and a power cost of 110,000 USD/MW using equation 25.[18]

$$CapEx_{Battery} = f_{Capacity} * S_{Battery} * tpd_{NH3} + f_{Power} * P_{Battery} \tag{25}$$

Where $f_{Capacity}$ and f_{Power} are the capacity and power cost factors for lithium-ion battery storage. The capacity factor of the battery ($S_{Battery}$) was calculated using the values from table S15 and the power of the battery ($P_{Battery}$) is equal to the power needed to drive the Haber-Bosch reactor and PSA system independently.

Finally, the capital cost for ammonia storage (equation 26) is calculated assuming a storage cost (f_{NH3}) of 810 USD/ton$_{NH3}$[15] and assuming that a fraction of the yearly capacity is stored ($frac_{NH3}$).

$$CAPEX_{NH3_{Storage}} = M_{NH3} * frac_{NH3} * f_{NH3} \tag{26}$$

7.5 'Black Box' Ammonia Reactor Model and Capital Cost Estimation

The energy expenditure of a 'Black Box' ammonia production system (E_{NH3}) can be calculated using equation 27.

$$E_{NH3} = \frac{\Delta G_{NH3}}{\eta_{NH3} * MM_{NH3}} \tag{27}$$

Where ΔG_{NH3} is the minimum theoretical energy required to produce a mole of ammo-

nia (338.7 kJ/mol_{NH3}), η_{NH3} is the energy efficiency of the ammonia production system (20% - 60%), and MM_{NH3} is the molar mass of ammonia (17 g/mol_{NH3}) normalized to kilograms per mole. Due to the uncertainty of future technologies, we keep this model as technology-agnostic as possible. However, we assume that there will still be a need for a purified nitrogen source. As such, we include the energy expenditure for nitrogen supply from a PSA system. The total energy expenditure for a 'Black Box' model is outlined in Table S16.

Table S16: 'Black Box' System Energy Expenditure

**Parameter**	**Abbreviation**	**Value**	**Normalized Value**
PSA	E_{PSA}	0.27 kWh/kg_{N2}	0.222 kWh/kg_{NH3}
NH_3	E_{NH3}	–	Equation 27 kWh/kg_{NH3}
Total	E_{Total}	–	$E_{PSA} + E_{NH3}$

The rated power consumption required by the 'Black Box' system can be calculated by multiplying the energy expenditure by the ammonia production rate (equation 28).

$$P_{BB} = \frac{kW_{DC} * E_{NH3}}{E_{Total}} \tag{28}$$

Then, the rated power consumption is used to calculate the capital cost for the 'Black Box' ammonia production system. The total capital costs related to the 'Black Box' ammonia system were calculated using a single power-scaling parameter (equation 29). This parameter is assumed to include the installation, offsite, design and engineering, and contingency cost due to the uncertainty of the technology's cost in the future and the fact that we are varying the parameter and studying the sensitivity of the parameter throughout the paper. Additionally, the capital cost for separations $CapEx_{Separations}$ was assumed to be 40,000 USD/tpd

$$CapEx_{BB} = C_{PEM} * P_{BB} + CapEx_{Separations} \tag{29}$$

The operation and maintenance cost of the 'Black Box' ammonia system depends on the capital cost and operation and maintenance factor ($OpEx_{BB}$).

$$OpEx_{BB} = O\&M_{PEM} * CapEx_{BB} \tag{30}$$

7.6 Solar Photovoltaic Cost Model and Capital Cost Estimation

For a facility of a given production capacity (M_{NH_3}) in kilograms per year, the ammonia production cost for a system powered by solar photovoltaic was calculated using equation 31-37. First, the energy required to operate a facility for a year is calculated using the values for table S13 for an electrified Haber-Bosch system and table S16 for a 'BlackBox' system using equation 31.

$$E_{Required} = E_{Total} * M_{NH3} \tag{31}$$

Where E_{Total} is the energy expenditure needed to produce one kilogram of ammonia and M_{NH3} is the total amount of ammonia produced in one year. We use $E_{Required}$ to calculate the total solar photovoltaic area and the rated peak power. The total solar photovoltaic area (A_{PV}) was calculated using equation 32.

$$A_{PV} = \frac{E_{Required}}{GTI * GCR * \eta_{PV}} \tag{32}$$

Where GCR is the ground coverage ratio which is the ratio between the module area over the required land area and is assumed to be 0.6,[19] GTI is the global tilted irradiance in $kJ/m^2 - year$, and η_{PV} is the solar panel efficiency calculated using equation 33-36. Then, we use the second dataset that describes the solar power potential (kWh/kWp) to calculate the maximum solar power input into the photovoltaic array (S in kW/m^2 – equation 33). We will use the maximum solar power input into the photovoltaic array (S) to calculate the average temperature and efficiency of the solar array and to size the solar

array and ammonia synthesis systems based on the peak power.

$$S = GTI/PV_{OUT} \tag{33}$$

Where GTI is the global tilted irradiance in $kWh/m^2{-}day$ and PV_{OUT} is the solar power potential (kWh/kWp). The solar power potential dataset excludes all locations where PV systems are not feasible due to physical or cultural constraints.

Then, we calculate the solar module temperature (equation 34) using the nominal operating cell temperature (NOCT) method, the average air temperature from the third dataset, and the calculated maximum power input (kW/m^2).

$$T_{Cell} = T_{Air} + \frac{NOCT - 20}{8,000} * S \tag{34}$$

Where NOCT is the nominal operating cell temperature and is assumed to be 48^oC for the average solar photovoltaic module,[20–22] T_{air} is the air temperature around the solar panel, and S is the maximum solar power input (S) in kW/m^2.

The solar module temperature (T_{Cell}), nominal solar panel efficiency, and the solar panel temperature coefficient ($\alpha_p = -0.31\%$) can be used to calculate the solar panel efficiency. This means that a 1°C increase in the solar panel temperature from ambient temperature (25°C) leads to a 0.31% decrease in solar panel efficiency. The correlation between efficiency and the solar panel temperature can be described using equation 35.

$$\eta_{PV} = \eta_o * (1 + \alpha_p * (T_{Cell} - 25)) \tag{35}$$

where η_o is the nominal solar panel efficiency (20%), T_{Cell} is the solar panel temperature, and α_p is the solar panel temperature coefficient (-0.31%). To calculate the peak power for a solar system (kW_{DC}) we multiply the maximum solar power input into the photovoltaic array (S), the area (A), the ground coverage ratio (GCR = 0.6), and the solar panel efficiency (η_{PV}) – equation 36.

$$kW_{DC_{PV}} = A_{PV} * GCR * S * \eta_{PV} \tag{36}$$

The peak power ($kW_{DC_{PV}}$) is used to size all other components in the system. The rated power for the PV system ($kW_{Rated_{PV}}$) is used to calculate the capital cost of the PV system and is calculated using equation 36 using standard conditions. As such, the maximum solar power input into the photovoltaic array (S) is assumed to be the average solar irradiance (S = 1 kW/m^2), and η_{PV} is assumed to be the nominal PV efficiency ($\eta_{PV} = 20\%$). The capital cost of the photovoltaic system is calculated using equation 37.

$$CapEx_{PV} = C_{PV} * kW_{Rated_{PV}} \tag{37}$$

Where C_{PV} is the overnight capital cost factor for solar photovoltaics in 2050. The operation and maintenance cost for the solar photovoltaic system depends on the peak power (equation 38).

$$OpEx_{PV} = O\&M_{PV} * kW_{DC_{PV}} \tag{38}$$

7.7 Wind Energy Model and Capital Cost Estimation

For a facility of a given production capacity (M_{NH_3}) in kilograms per year, the ammonia production cost for a system powered by wind energy was calculated using equation 39-42. First, the energy required to operate a facility for a year is calculated using the values for table S13 for an electrified Haber-Bosch system and table S16 for a 'BlackBox' system using equation 39.

$$E_{Required} = E_{Total} * M_{NH3} \tag{39}$$

Where E_{Total} is the energy expenditure needed to produce one kilogram of ammonia and M_{NH3} is the total amount of ammonia produced in one year. We use E_{Total} to cal-

culate the total solar photovoltaic area and the rated peak power. The maximum power produced by the wind farm in any location depends on the power density of wind power and is described in equation 40.

$$kW_{DC_{Wind}} = \frac{E_{Required}}{CF * 365 * 24} \tag{40}$$

where CF is the capacity factor for wind, the rated power for the wind system ($kW_{DC_{Wind}}$) is used to calculate the capital cost of the wind system and is equal to the maximum power produced by the wind farm in equation 40. The capital cost of the wind system is calculated using equation 41.

$$CapEx_{Wind} = C_{Wind} * kW_{DC_{Wind}} \tag{41}$$

The operation and maintenance cost for the wind system depends on the peak power and the total energy produced in a year (equation 42).

$$OpEx_{Wind} = O\&M_{Wind} * kW_{DC_{Wind}} + O\&M_{Var} * E_{Required} \tag{42}$$

8 Calculating Fertilizer Demand

The spatial distribution of the fertilizer demand correlates to the spatial distribution of the global croplands (Fig. 6).[1] This dataset has a resolution of 1 km at the equator and was sorted to identify all pixels corresponding to croplands. However, to convert these binary pixels into the amount of fertilizer (ammonia) needed we had to calculate the actual area corresponding to each pixel using equations 2 to 4 with a pixel resolution of $\sim$ 0.009 degrees. The input fertilizer into each pixel was calculated assuming a fertilizer input of 100 $kg_N/ha{*}yr$[23] and normalized to ammonia using the molecular weights of nitrogen and ammonia. A fertilizer input of 100 $kg_N/ha{*}yr$ is assumed to be the average recommended cropland input. However, the fertilizer input will likely change depending on the crop type, location, and agriculture practices. Changing the cropland nitrogen input per area does not affect the optimal location of the photovoltaic-driven ammonia production facilities and will only affect the size of these facilities. So, a fertilizer input of 200 $kg_N/ha * yr$, will result in production facilities at the same optimal locations but with twice the size. Finally, the resolution of the dataset was decreased from 1km to around 10km sized pixels by adding each pixel to the nearing 0.1 degrees.

Where the area for each location in the grid (A) was calculated using equations 43-45.

$$w = \Delta deg * w_o * cos(lat) \tag{43}$$

$$h = \Delta deg * h_o \tag{44}$$

$$A = w * h \tag{45}$$

Where A is the area for each location in the grid (km^2), w and h are the width and height of each location in the grid (km), Δdeg is the resolution of the model in degrees ($\sim$

0.1 degrees), w_o is the length of one-degree longitude at the equator (w_o = 111.32 km), h_o is the length of one-degree latitude at the equator (h_o = 110.574 km), and lat and lon are the latitude and longitude at each location.

9 Multi-objective Optimization

In order to calculate the optimal distribution network we used an exhaustive search algorithm that aims to minimize a score function (equation 23). This means that for every farm we surveyed every possible ammonia production location and selected the one that resulted in the lowest score.

$$Score = w_1 * (LCOA_{NH3} + C_t * d_t) + w_2 * WS \tag{46}$$

Where $LCOA_{NH3}$ is the ammonia production cost (i.e. levelized cost of ammonia) at the production location, C_t is the ammonia transportation cost in USD/ton$_{NH3}$-km, d_t is the transportation distance between the production facility and the farms, WS is the water stress at the production location, w_1 is the weight placed on the ammonia cost and w_2 is the weight placed on the water stress. These weights signify the relative importance placed on cost and water. For example, a scenario that prioritizes cost has weights w_1 equal to one and w_2 equal to zero. This means that all the importance is placed on minimizing the cost. On the other hand, a scenario that prioritizes water has weights $w_1 = 0.01$ and $w_2 = 0.99$. This means that the optimization score is composed of 1% by the ammonia cost and 99% by the water stress. The exhaustive optimization algorithm minimizes the score in equation 46 for every possible farm to find the optimal production location for all farms. The transportation cost (C_t) is assumed to be 0.016 USD/ton$_{NH3}$-km for transportation by ship, 0.04 USD/ton$_{NH3}$-km for transportation by pipeline, and 0.09 USD/ton$_{NH3}$-km for transportation by truck.[4] For the baseline scenario, we used a transportation cost of 0.09 USD/ton$_{NH3}$-km. Finally, the distribution distance (d_t) between the production facilities and the farms is calculated using the haversine formula (equation 47).

$$d_t = 2 * R_{Earth} * arcsin(\sqrt{sin^2(\frac{lat_2 - lat_1}{2}) + cos(lat_1) * cos(lat_2) * sin^2(\frac{lon_2 - lon_1}{2})} \quad (47)$$

Where R_{Earth} is the radius of earth in kilometers (R_{Earth} = 6,373 km), lat_1 and lon_1 are the coordinates of the prospective production location, and lat_2 and lon_2 are the coordinates of the farm.

10 Methane-Fed Haber-Bosch Process Production Cost Model

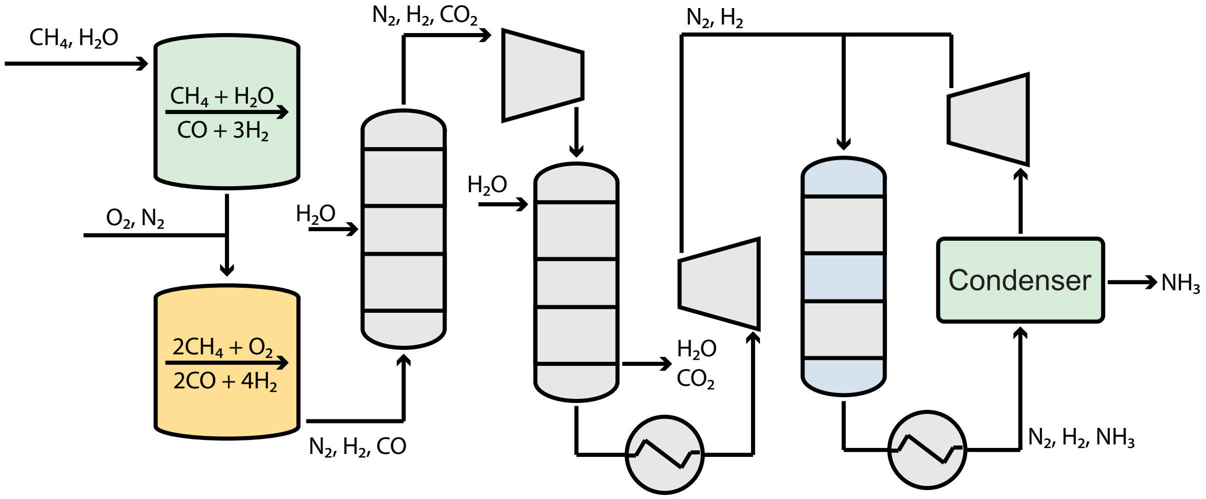

Figure 1: Methane-fed Haber-Bosch process diagram.

The capital cost and energy expenditure of a methane-fed Haber-Bosch ammonia process (HB) are calculated based on the ammonia production capacity. The Haber-Bosch process was modeled in ASPEN Plus following the flowsheet shown in Figure S1. This process consists of two key elements: a hydrogen production loop and an ammonia synthesis loop (Figure S1). In the hydrogen production loop, two steam methane reforming reactors are employed to produce hydrogen from methane. The primary steam methane reforming reactor facilitates the conversion of methane and water into carbon monoxide and hydrogen. The secondary steam methane reforming reactor combines the remaining methane with air, enabling the conversion of methane into carbon monoxide and hydrogen while simultaneously eliminating oxygen from the air. The resulting carbon monoxide and remaining water are then directed to a water gas shift reactor, where they are transformed into carbon dioxide and hydrogen. Subsequently, the carbon dioxide is removed through the Benfield or Selexol method and any remaining carbon monoxide and carbon dioxide

are converted back into methane in a methanation reactor to minimize catalyst poisoning. Similarly, the ammonia synthesis loop includes a Haber-Bosch reactor and a separation train. These components operate at elevated temperatures and pressures, necessitating the utilization of expensive equipment and systems.

From the ASPEN Plus simulation, we calculated the ammonia produced, natural gas consumed, water consumed, and electricity consumed as well as individual component sizing at different scales. The material inputs and outputs required for a methane-fed Haber-Bosch facility are outlined in Table S17.

Table S17: Material flow for the methane-fed Haber-Bosch process

**Material Inputs**	**Value –** kg/kg_{NH3}
Natural Gas (Furnace)	0.153
Natural Gas (Reformer)	0.543
Air (Furnace)	2.67
Air (Reformer)	1.13
Steam	336
**Material Outlets**	**Value –** kg/kg_{NH3}
CO_2 (Furnace)	0.381
CO_2 (Reformer)	1.18

The natural gas input for the reformer enters the system at 45 degrees Celsius and is heated up to 345 degrees Celsius before sulfur is removed. We modeled the desulfuration by combining an RStoic and Sep2 blocks to remove all the sulfur from the input natural gas stream. Then, the sulfur-free output is combined with 1.81 kg/kg_{NH3} of high-pressure steam at (T = 360°C and P = 35.3 bar). The natural gas and steam mixture is then heated up to 502 degrees Celsius. The natural gas and steam enter the primary reformer which consists of an RStoic and RGibbs reactor. Higher-order hydrocarbons are reformed rapidly in the RStoic reactor and methane is reformed in the RGibbs reactor. Then, air is added at 1.13 kg/kg_{NH3} to the outlet of the primary reformer, compressed to 32.4 bar in a multi-stage compressor, and then heated to 465 degrees Celsius. The heated compressed air and the output from the primary reformer enter a secondary reformer modeled

using an RGibbs reactor with zero heat duty. The outlet from the secondary reformer enters the series of high-temperature and low-temperature water gas shift reactors for CO removal which are modeled with two RStoic reactors with zero heat duty and temperatures inlet temperatures of 380 degrees Celsius and 210 degrees Celsius. The output is cooled down to 40 degrees Celsius before CO2 is removed using an ammonia adsorption process. The adsorber operates at 40 degrees Celsius and the desorption column operates at 100 degrees Celsius. Methanation takes place in an RStoic reactor with zero duty and an inlet temperature of 240 degrees Celsius where CO2 and CO2 are converted to methane. Then, water is removed and the outlet is compressed to 275 bar before entering the Haber-Bosch reactors. The system sizing and conditions for each component are outlined in Tables S18 and S19.

The wall thickness (t_w) of all pressurized reactors was calculated from the diameter and the design pressure (48).

$$t_w = \frac{P_i * D_c}{2 * SE - 1.2 * P_i} \tag{48}$$

where P_i is the design pressure (which is 10% higher than the operating pressure in N/m^2), D_c is the column diameter in meters, and SE is the maximum allowable stress for 304 stainless steel at the operating temperature. From the wall thickness, length, and diameter, we can calculate the reactor shell mass using equation 49.

$$SM = \pi * D_c * L_c * t_w * \rho \tag{49}$$

where D_c is the diameter in meters, L_c is the length in meters, t_w is the wall thickness in meters, and ρ is the density for 304 stainless steel (ρ = 8,000 kg/m^3). The compressor power, heat exchanger area, and the absorbers' shell mass are used to calculate the uninstalled capital cost for the methane-fed Haber-Bosch system in equations 50.

Table S18: Methane-fed Haber-Bosch System Sizing for 1,350 tpd_{NH3} capacity from ASPEN Plus model.

**Component**	**Size**				
Furnace (MW)	72				
Compressors (kW)	1357.5	1434.8	1437.9	1443.8	8818.3
Compressors (kW)	3925.3	4049.9	4289.4	1531.8	
Heat Exchangers (m^2)	753	753	753	84.1	601.7
Heat Exchangers (m^2)	183.5	168.9	31.9	15.8	547.3
Heat Exchangers (m^2)	21.1	53.8	103	205.1549105	132
Heat Exchangers (m^2)	31.1	101.6	226.9	12.5	396.9
Heat Exchangers (m^2)	98.7	335.7	181.3		
Primary Reformer Volume (m^3)	0.15				
Primary Reformer Ratio (L/D)	71				
Primary Reformer Pressure (bar)	32.4				
Primary Reformer Temperature ($^{\circ}C$)	502				
Secondary Reformer Volume (m^3)	33.22				
Secondary Reformer Ratio (L/D)	1.6				
Secondary Reformer Pressure (bar)	31.4				
Secondary Reformer Temperature ($^{\circ}C$)	800				
LT WGS Volume (m^3)	82.8				
LT WGS Reformer Ratio (L/D)	2				
LT WGS Reformer Pressure (bar)	31.4				
LT WGS Reformer Temperature ($^{\circ}C$)	210				
HT WGS Volume (m^3)	60.1				
HT WGS Reformer Ratio (L/D)	7.2				
HT WGS Reformer Pressure (bar)	31.4				
HT WGS Reformer Temperature ($^{\circ}C$)	380				
Absorber Volume (m^3)	65.6				
Absorber Reformer Ratio (L/D)	2.95				
Absorber Reformer Pressure (bar)	33.8				
Absorber Reformer Temperature ($^{\circ}C$)	40				
Stripper Volume (m^3)	92.1				
Stripper Reformer Ratio (L/D)	2.12				
Stripper Reformer Pressure (bar)	1				
Stripper Reformer Temperature ($^{\circ}C$)	100				

$$C = a + b * S^n \tag{50}$$

Table S19: Methane-fed Haber-Bosch System Sizing for 1,350 tpd_{NH3} capacity from ASPEN Plus model (cont.).

**Component**	**Size**				
Flash Drum Volume (m^3)	11.3	2.4	17.9	17.9	18.3
Flash Drum Reformer Ratio (L/D)	1.85	4	2.92	2.92	3
Flash Drum Reformer Pressure (bar)	29	32.4	287.7	32.4	287.7
Flash Drum Volume (m^3)	6.7	11.3			
Flash Drum Reformer Ratio (L/D)	2.4	1.8			
Flash Drum Reformer Pressure (bar)	287.7	287.7			
Methanizer Volume (m^3)	0.03				
Methanizer Ratio (L/D)	27.3				
Methanizer Pressure (bar)	25.5				
Methanizer Temperature ($°C$)	240				
Desulfurator Volume (m^3)	35.8				
Desulfurator Ratio (L/D)	3.75				
Desulfurator Pressure (bar)	34.3				
Desulfurator Temperature ($°C$)	15				
Haber-Bosch Reactors	#1	#2	#3	#4	
Inlet Pressure (bar)	292	290	288	286	
Inlet Temperature ($°C$)	445	460	410	345	
Catalyst Mass (kg)	8,902	9,712	13,353	29,135	
Reactor Volume (m^3)	2.74	2.99	4.11	8.96	
Reactor Aspect Ratio (L/D)	2	2	2	2	

The values for each one of the components of this equation for the Haber-Bosch synthesis system are highlighted in Table S20.

Table S20: Cost Scaling Parameters for the methane-fed Haber-Bosch.[3]

**Equipment**	**Units for S**	**a**	**b**	**n**
Furnace	heat duty (MW)	80,000	109,000	0.8
Centrifugal Compressor	driver power (kW)	580,000	20,000	0.6
U-tube shell and tube Heat Exchanger	area (m^2)	28,000	54	1.2
Vertical Pressure Vessel (304 ss)	shell mass (kg)	17,400	79	0.85

The installed capital cost for each parameter is calculated by multiplying the uninstalled capital cost by the installation factor (Equation 51 and Table S21).

$$C_i = f_i * C \tag{51}$$

Table S21: Installation Factors for the methane-fed Haber-Bosch.[3]

Equipment	Installation Factor
Furnace	2
Centrifugal Compressor	2.5
Vertical Pressure Vessel (304 ss)	4
Heat Exchanger	3.5

The installed capital cost represents the inside battery limit (ISBL) costs. Additional costs, referred to as the outside battery limit (OSBL) costs are assumed to be 30% of the ISBL costs. Additional design and engineering costs are 30% of the total plant costs (ISBL + OSBL) and contingency costs are 10% of the total plant costs (ISBL + OSBL). As such, the total capital costs related to the PSA system are 1.82 times the ISBL costs after adding OSBL, design and engineering, and contingency (equation 52).

$$CapEx_{mHB} = 1.82 * C_i \tag{52}$$

Additionally, the methane-fed Haber-Bosch process requires 0.7 kilograms of natural gas per kilogram of ammonia produced and 1,810 kJ of electricity per kilogram of ammonia produced. Yearly costs amounting to maintenance, insurance, and land were estimated to be 4%, 2%, and 2% of the total overnight capital cost.

The ammonia production cost – or levelized cost of ammonia – is a function of the discounted sum of the yearly costs over the discounted sum of the yearly ammonia produced for the entire lifetime of the project. The ammonia production cost – or levelized cost of ammonia – can be calculated using equation 7.

$$LCOA_{NH3} = \frac{CapEx_{mHB} + \sum_{t=0}^{t=lifetime} \frac{OpEx_{mHB,t}}{(1+d)^t}}{\sum_{t=0}^{t=lifetime} \frac{M_{NH3}}{(1+d)^t}} \tag{53}$$

Where CapEx is the initial capital investment, OpEx is the yearly operation costs, d is the discount rate, t is the year, and M_{NH3} is the yearly ammonia production.

11 Supporting Tables

Table S22: Results from Figure 2. Average average geographic cost of ammonia production.

Technology	Low-Capital Cost	High-Capital Cost
Electrified Haber-Bosch	870 USD/t_{NH3}	1,600 USD/t_{NH3}
Low-Efficiency 'Black Box'	1,500 USD/t_{NH3}	3,700 USD/t_{NH3}
Medium-Efficiency 'Black Box'	800 USD/t_{NH3}	1,900 USD/t_{NH3}
High-Efficiency 'Black Box'	570 USD/t_{NH3}	1,300 USD/t_{NH3}

Table S23: Results from Figure 2. Natural gas prices and economic viability.

Technology	Low-Capital Cost	High-Capital Cost
Electrified Haber-Bosch	18 $USD/MMBtu$	39 $USD/MMBtu$
Low-Efficiency 'Black Box'	36 $USD/MMBtu$	90 $USD/MMBtu$
Medium-Efficiency 'Black Box'	17 $USD/MMBtu$	47 $USD/MMBtu$
High-Efficiency 'Black Box'	10 $USD/MMBtu$	30 $USD/MMBtu$

Table S24: Results from Figure 3. The optimal number of regional production locations.

Technology	Low-Capital Cost	High-Capital Cost
Electrified Haber-Bosch	144	78
Low-Efficiency 'Black Box'	124	32
Medium-Efficiency 'Black Box'	329	92
High-Efficiency 'Black Box'	795	164

Table S25: Results from Figure 3. Optimal ammonia production cost.

Technology	Low-Capital Cost	High-Capital Cost
Electrified Haber-Bosch	707 USD/t_{NH3}	1,015 USD/t_{NH3}
Low-Efficiency 'Black Box'	1,000 USD/t_{NH3}	1,800 USD/t_{NH3}
Medium-Efficiency 'Black Box'	560 USD/t_{NH3}	1,000 USD/t_{NH3}
High-Efficiency 'Black Box'	410 USD/t_{NH3}	710 USD/t_{NH3}

Table S26: Results from Figure 3. Optimal ammonia distribution cost.

Technology	Low-Capital Cost	High-Capital Cost
Electrified Haber-Bosch	43 USD/t_{NH3}	75 USD/t_{NH3}
Low-Efficiency 'Black Box'	90 USD/t_{NH3}	230 USD/t_{NH3}
Medium-Efficiency 'Black Box'	55 USD/t_{NH3}	110 USD/t_{NH3}
High-Efficiency 'Black Box'	41 USD/t_{NH3}	80 USD/t_{NH3}

Table S27: Results from Figure 3. Optimal ammonia distribution distance.

Technology	Low-Capital Cost	High-Capital Cost
Electrified Haber-Bosch	480 km	840 km
Low-Efficiency 'Black Box'	1,000 km	2,500 km
Medium-Efficiency 'Black Box'	600 km	1,200 km
High-Efficiency 'Black Box'	460 km	880 km

Table S28: Results from Figure 4a. Effect of discount rate on the ammonia cost.

Discount Rate	Low-Capital Cost	High-Capital Cost
3%	540 USD/t_{NH3}	950 USD/t_{NH3}
7%	620 USD/t_{NH3}	1,100 USD/t_{NH3}
10%	680 USD/t_{NH3}	1,230 USD/t_{NH3}

Table S29: Results from Figure 4b. Effect of discount rate on the average distribution distance.

Discount Rate	Low-Capital Cost	High-Capital Cost
3%	510 km	1,020 km
7%	600 km	1,200 km
10%	660 km	1,400 km

Table S30: Results from Figure 4c. Effect of discount rate on the number of production regions.

Discount Rate	Low-Capital Cost	High-Capital Cost
3%	1,015	127
7%	329	92
10%	241	75

Table S31: Results from Figure 4d. Effect of discount rate on the average production capacity.

Discount Rate	Low-Capital Cost	High-Capital Cost
3%	950 tpd_{NH3}	7,600 tpd_{NH3}
7%	2,900 tpd_{NH3}	10,500 tpd_{NH3}
10%	4,000 tpd_{NH3}	13,000 tpd_{NH3}

Table S32: Results from Figure 5. Wind and photovoltaic electricity-driven ammonia and water uncertainty.

Parameter	Prioritizing Cost	Prioritizing Water
Production Cost (USD/t_{NH3})	805	822
Distribution Cost (USD/t_{NH3})	85	82
Water Stress	6.8	0.08

12 Supporting Figures

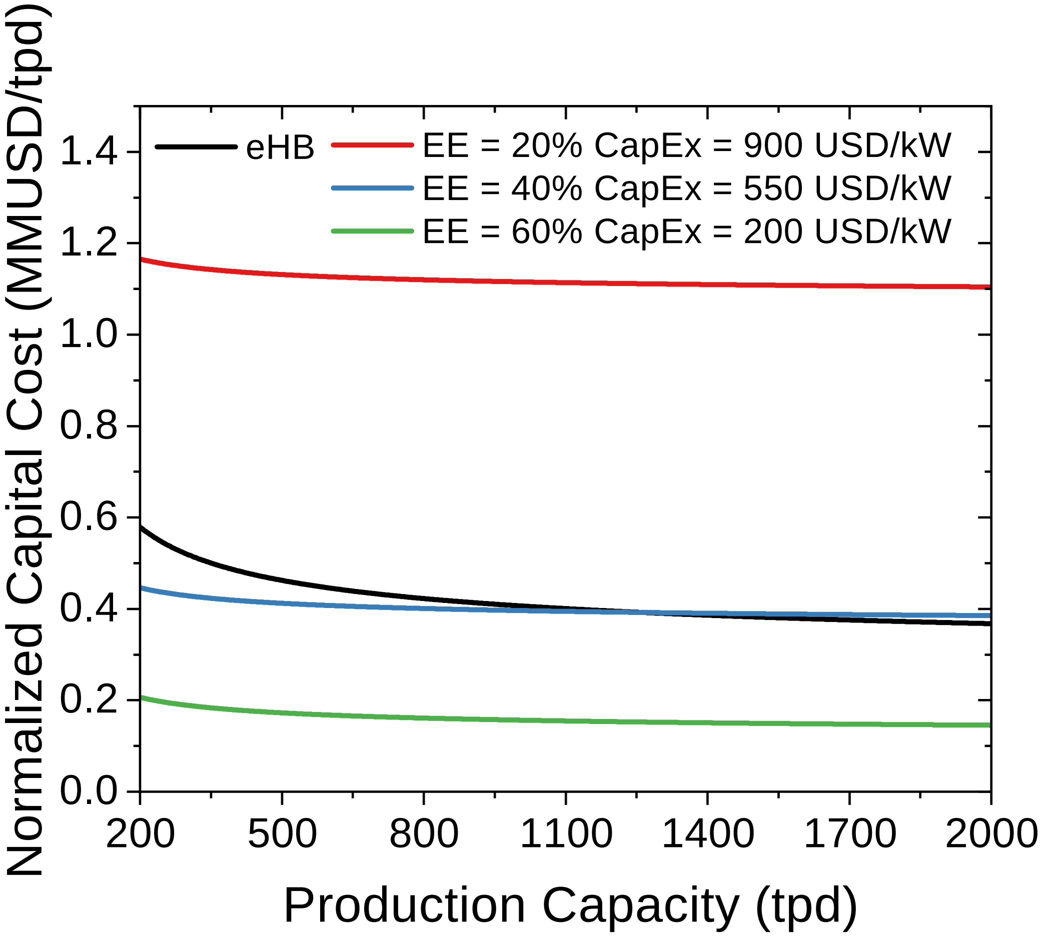

Figure 2: Normalized capital cost of different technology scenarios with variations in production capacity.

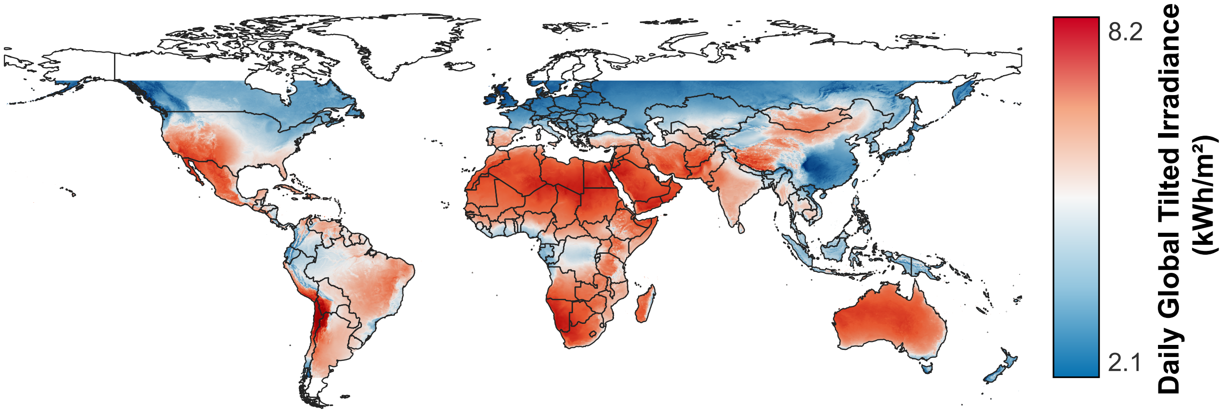

Figure 3: Average daily global tilted irradiance at the optimal tilt angle obtained from the Global Solar Atlas 2.0, a free, web-based application is developed and operated by the company Solargis s.r.o. on behalf of the World Bank Group, utilizing Solargis data, with funding provided by the Energy Sector Management Assistance Program (ESMAP). For additional information: https://globalsolaratlas.info

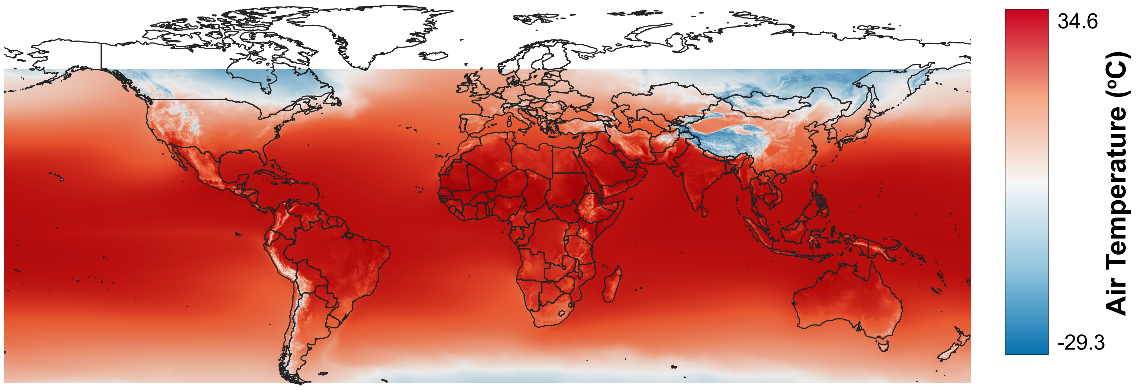

Figure 4: Air Temperature at 2 m above ground level obtained from the Global Wind Atlas 3.0, a free, web-based application developed, owned and operated by the Technical University of Denmark (DTU). The Global Wind Atlas 3.0 is released in partnership with the World Bank Group, utilizing data provided by Vortex, using funding provided by the Energy Sector Management Assistance Program (ESMAP). For additional information: https://globalwindatlas.info

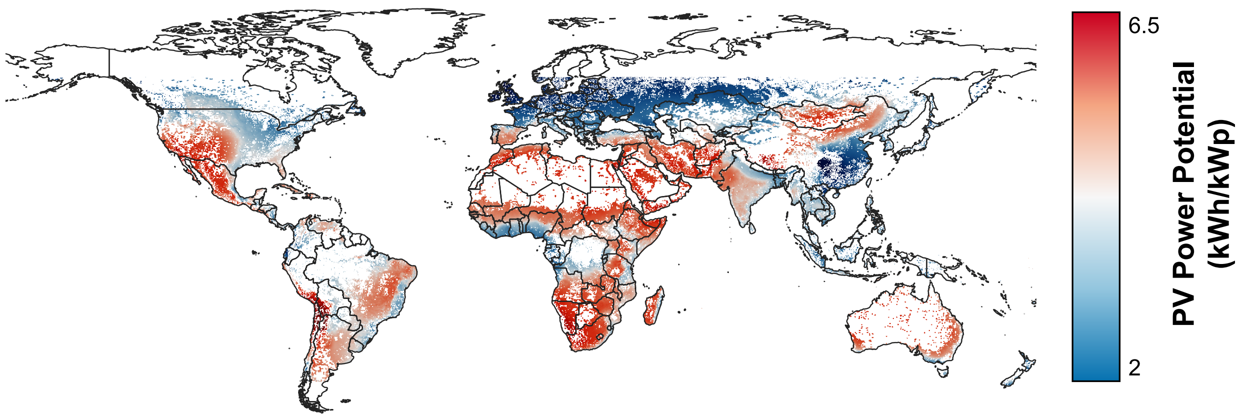

Figure 5: Level 1 practical PV potential obtained from the Global Solar Atlas 2.0, a free, web-based application is developed and operated by the company Solargis s.r.o. on behalf of the World Bank Group, utilizing Solargis data, with funding provided by the Energy Sector Management Assistance Program (ESMAP). For additional information: https://globalsolaratlas.info

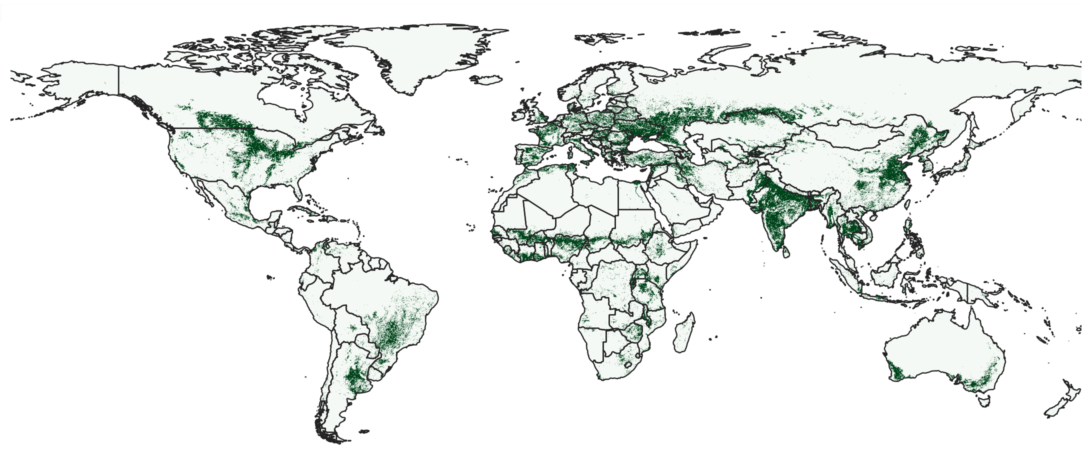

Figure 6: Locations of global agriculture centers.[1]

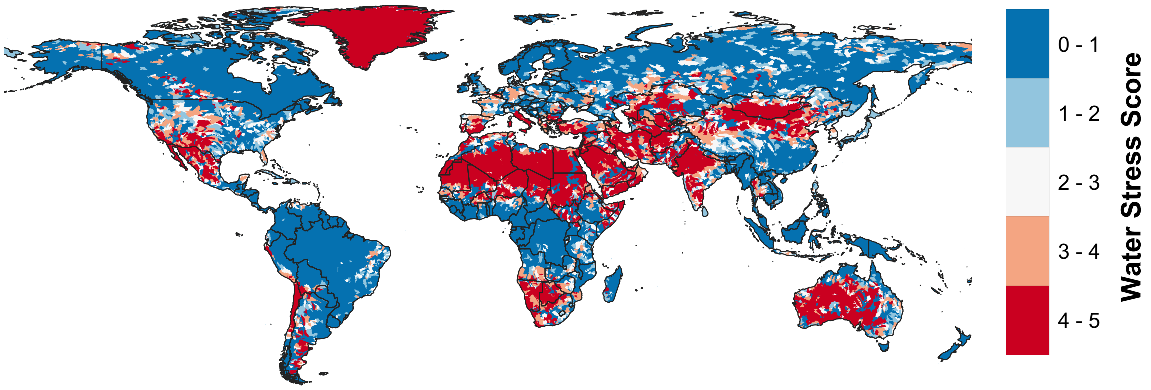

Figure 7: Water Stress Score. Source: WRI Aqueduct. Source: aqueduct.wri.org.

References

(1) Thenkabail, P.; Teluguntla, P.; Xiong, J.; Oliphant, A.; Massey, R. NASA MEaSUREs Global Food Security-Support Analysis Data (GFSAD) Crop Mask 2010 Global 1 km V001. *NASA EOSDIS Land Processes DAAC* **2016**,

(2) Marcinek, A.; Möller, A.; Guderian, J.; Bathen, D. Dynamic simulation of high-purity twin-bed N2-PSA plants. *Adsorption* **2021**, *27*, 1149–1173.

(3) Towler, G.; Sinnott, R. *Chemical engineering design: principles, practice and economics of plant and process design*; Butterworth-Heinemann, 2021.

(4) IEA, The Future of Hydrogen. *IEA* **2019**,

(5) Tripodi, A.; Compagnoni, M.; Bahadori, E.; Rossetti, I. Process simulation of ammonia synthesis over optimized Ru/C catalyst and multibed Fe+ Ru configurations. *Journal of Industrial and Engineering Chemistry* **2018**, *66*, 176–186.

(6) Bose, A.; Lazouski, N.; Gala, M.; Manthiram, K.; Mallapragada, D. Spatial variation in cost of electricity-driven continuous ammonia production in the United States. **2022**,

(7) Program, E. S. M. A. *Global photovoltaic power potential by country*; World Bank, 2020.

(8) Program, E. S. M. A. *The Global Wind Atlas 3.0*; World Bank, 2022.

(9) Kuzma, S.; Bierkens, M. F.; Lakshman, S.; Luo, T.; Saccoccia, L.; Sutanudjaja, E. H.; Van Beek, R. Aqueduct 4.0: Updated Decision-Relevant Global Water Risk Indicators. **2023**,

(10) EIA, Annual energy outlook 2022. *Energy Information Administration, Washington, DC* **2022**,

(11) Tran, T. T.; Smith, A. D. Incorporating performance-based global sensitivity and uncertainty analysis into LCOE calculations for emerging renewable energy technologies. *Applied energy* **2018**, *216*, 157–171.

(12) Pascual, J.; Martinez-Moreno, F.; García, M.; Marcos, J.; Marroyo, L.; Lorenzo, E. Long-term degradation rate of crystalline silicon PV modules at commercial PV plants: an 82-MWp assessment over 10 years. *Progress in Photovoltaics: Research and Applications* **2021**, *29*, 1294–1302.

(13) Staffell, I.; Green, R. How does wind farm performance decline with age? *Renewable energy* **2014**, *66*, 775–786.

(14) Fernandez, C. A.; Hatzell, M. C. Editors' Choice—Economic Considerations for Low-Temperature Electrochemical Ammonia Production: Achieving Haber-Bosch Parity. *Journal of The Electrochemical Society* **2020**, *167*, 143504.

(15) Valera-Medina, A.; Banares-Alcantara, R. *Techno-economic challenges of green ammonia as an energy vector*; Academic Press, 2020.

(16) Smith, B. L. *Updates to the Instant Online PV LCOE Calculator Tool*; 2021.

(17) Palys, M. J.; Daoutidis, P. Using hydrogen and ammonia for renewable energy storage: A geographically comprehensive techno-economic study. *Computers & Chemical Engineering* **2020**, *136*, 106785.

(18) Akar, S.; Beiter, P.; Cole, W.; Feldman, D.; Kurup, P.; Lantz, E.; Margolis, R.; Oladosu, D.; Stehly, T.; Rhodes, G., et al. *2020 annual technology baseline (ATB) cost and performance data for electricity generation technologies*; 2020.

(19) Dobos, A. P. PVWatts Version 5 Manual.

(20) Sun, V.; Asanakham, A.; Deethayat, T.; Kiatsiriroat, T. Evaluation of nominal operating cell temperature (NOCT) of glazed photovoltaic thermal module. *Case Studies in Thermal Engineering* **2021**, *28*, 101361.

(21) Ross, R. G., Jr; Smokler, M. I. Flat-Plate Solar Array Project: Final report: Volume 6, Engineering sciences and reliability.

(22) Ross Jr, R. Flat-plate photovoltaic array design optimization. 14th Photovoltaic Specialists Conference. 1980; pp 1126–1132.

(23) Comer, B. M.; Fuentes, P.; Dimkpa, C. O.; Liu, Y.-H.; Fernandez, C. A.; Arora, P.; Realff, M.; Singh, U.; Hatzell, M. C.; Medford, A. J. Prospects and challenges for solar fertilizers. *Joule* **2019**, *3*, 1578–1605.
